# Characterization of global transcription profile of normal and HPV-immortalized keratinocytes and their response to TNF treatment

**DOI:** 10.1186/1755-8794-1-29

**Published:** 2008-06-27

**Authors:** Lara Termini, Enrique Boccardo, Gustavo H Esteves, Roberto Hirata, Waleska K Martins, Anna Estela L Colo, E Jordão Neves, Luisa Lina Villa, Luiz FL Reis

**Affiliations:** 1 Ludwig Institute for Cancer Research, São Paulo, Brazil; 2 Hospital do Câncer A. C. Camargo, São Paulo, Brazil; 3 Instituto de Matemática e Estatística da Universidade de São Paulo, São Paulo, Brazil

## Abstract

**Background:**

Persistent infection by high risk HPV types (e.g. HPV-16, -18, -31, and -45) is the main risk factor for development of cervical intraepithelial neoplasia and cervical cancer. Tumor necrosis factor (TNF) is a key mediator of epithelial cell inflammatory response and exerts a potent cytostatic effect on normal or HPV16, but not on HPV18 immortalized keratinocytes. Moreover, several cervical carcinoma-derived cell lines are resistant to TNF anti-proliferative effect suggesting that the acquisition of TNF-resistance may constitute an important step in HPV-mediated carcinogenesis. In the present study, we compared the gene expression profiles of normal and HPV16 or 18 immortalized human keratinocytes before and after treatment with TNF for 3 or 60 hours.

**Methods:**

In this study, we determined the transcriptional changes 3 and 60 hours after TNF treatment of normal, HPV16 and HPV18 immortalized keratinocytes by microarray analysis. The expression pattern of two genes observed by microarray was confirmed by Northern Blot. NF-κB activation was also determined by electrophoretic mobility shift assay (EMSA) using specific oligonucleotides and nuclear protein extracts.

**Results:**

We observed the differential expression of a common set of genes in two TNF-sensitive cell lines that differs from those modulated in TNF-resistant ones. This information was used to define genes whose differential expression could be associated with the differential response to TNF, such as: *KLK7 *(*kallikrein 7*), *SOD2 *(*superoxide dismutase 2*), *100P *(*S100 calcium binding protein P*), *PI3 *(*protease inhibitor 3, skin-derived*), *CSTA *(*cystatin A*), *RARRES1 *(*retinoic acid receptor responder 1*), and *LXN *(*latexin*). The differential expression of the *KLK7 *and *SOD2 *transcripts was confirmed by Northern blot. Moreover, we observed that *SOD2 *expression correlates with the differential NF-κB activation exhibited by TNF-sensitive and TNF-resistant cells.

**Conclusion:**

This is the first in depth analysis of the differential effect of TNF on normal and HPV16 or HPV18 immortalized keratinocytes. Our findings may be useful for the identification of genes involved in TNF resistance acquisition and candidate genes which deregulated expression may be associated with cervical disease establishment and/or progression.

## Background

Human papillomaviruses (HPVs) are double-stranded DNA tumor viruses that infect keratinocytes of the anogenital tract epithelium [1]. Persistent infection by high risk HPV types (e.g., HPV-16, -18, -31, and -45) is the main risk factor for the development of cervical intraepithelial neoplasia and cervical cancer [2,3]. High-risk HPV DNA is detected in more than 90% of cervical carcinomas worldwide [4] and it has been shown that HPV types 16 and 18 can immortalize normal cells in culture, a function that is attributed to E6 and E7 oncogenes [5]. These are the only HPV genes consistently retained and expressed in cervical carcinomas. Besides, their continued expression is required to maintain the malignant phenotype [6-8]. The proteins encoded by these genes disturb cell proliferation and differentiation by physical and functional interaction with several cellular factors involved in cell cycle regulation [9]. E6 is best known for its ability to bind to p53 and induce its ubiquitin-dependent degradation [10,11], whereas E7 was initially recognized by its ability to interact with members of the retinoblastoma protein family, namely pRb, p107 and p130 [12] and its capacity of enhancing their degradation [13].

Persistence of HPV infections and development of neoplasia is influenced by local cell-mediated immune response [14]. Tumor necrosis factor-alpha (TNF) is one of the main mediators of skin and mucosa inflammation and has a potent antiproliferative effect on normal primary human keratinocytes (PHKs). This cytokine is a key regulator of diverse inflammatory and immune processes in human epithelia and its expression by keratinocytes is enhanced in response to tissue injury, inflammation, viral infection, and UV radiation [15-17]. Furthermore, TNF has been identified as a key mediator for the regression of HPV-induced lesions [18-21]. Previous studies from our group had shown that TNF exerts a potent cytostatic effect on normal and HPV16 immortalized keratinocytes. On the other hand, keratinocytes immortalized by HPV18 or SV40, as well as HPV16 or HPV18-positive cervical tumor-derived cell lines continue to proliferate normally in the presence of this cytokine [22,23]. In addition, it has been observed that continuous HPV18-gene expression in malignant HeLa-fibroblasts hybrids, as well as increased tumorigenicity of HPV16-transformed human keratinocytes is associated with TNF resistance [24,25]. These observations underscore the importance of TNF-resistance acquisition in HPV mediated pathogenesis and suggest that this event could be an important factor in HPV-associated neoplasia outcome. However, the molecular basis of HPV-mediated TNF resistance has not been elucidated.

The aim of the present study was to characterize and compare the global transcription profile of normal and HPV-immortalized keratinocytes. Furthermore, we sought to analyze their response to TNF in order to identify differences that contribute to explain their divergent response to this cytokine. For this purpose, we used microarray analysis to determine transcriptional changes upon 3 and 60 hours after TNF exposure. The 3 hours treatment would favor the identification of immediate early TNF regulated genes. On the other hand, the 60 hours treatment was used because the cytostatic effect exerted by this cytokine on normal and HPV16-immortalized keratinocytes reaches its maximum at this time-point [22,23]. Our experimental setting allowed us to: 1) identify genes that are differentially expressed between TNF-sensitive and TNF-resistant cells; 2) identify genes that are differentially modulated by TNF at two-time points (3 and 60 hours); 3) analyze the effect of HPV-induced immortalization on TNF-regulated genes and, 4) find genes that are differentially expressed between cells immortalized by two different high-risk HPV types. Using this approach, we identified differentially expressed genes that are involved in different cell processes such as immune and inflammatory responses, cell differentiation, cell death, proliferation, extracellular matrix remodeling and DNA repair. The implications of these results are discussed.

## Methods

### Cell Culture and TNF treatment

Cultures of primary human keratinocytes (PHK), recovered from newborn foreskin (Cambrex, Walkersville, MD, USA), were maintained in keratinocyte serum-free medium – KSFM (Life Technology, Inc., Gaithersburg, MD, USA) for 3 to seven passages [26]. HF698 and HF18Nco are cell lines obtained from human keratinocytes immortalized by HPV16 and HPV18 whole genome, respectively. These cell lines (from now on referred as HPV16 and HPV18, respectively) were kindly provided by R. Schlegel, Georgetown University Medical Center, Washington, DC [27], were grown in 3+1 medium, consisting of a mixture of 3 parts KSFM and 1 part DMEM, supplemented with 10% fetal calf serum. Cells were grown in 100-mm tissue culture dishes to 30% confluence and treated with 2 nM of human recombinant TNF (Boehringer Mannheim, Germany), for 3 or 60 hours. Cells were then trypsinized, washed 3 times with PBS and frozen until RNA extraction. For all time points, RNA was obtained from two independent experiments, including the control plates.

### RNA extraction, amplification, labeling, and hybridizations

For each sample, total RNA was extracted using TRIzol Reagent (Life Technologies, Inc., Grand Island, NY, USA) following the procedure recommended by the manufacturer. Three micrograms of target and reference (a pool of RNA from all control conditions) total RNA were linearly amplified using T7-based protocol, converted to cyanine-modified cDNA, and labeled as described previously [28].

Hybridizations were performed in duplicate, using dye-swap, on a cDNA platform of ORESTES representing 4,600 unique genes with known full-length sequence selected from the clone collection derived from the Human Cancer Genome Project [29]. cDNA amplification, purification, identity verification and printing were performed as previously described [28]. A detailed description of the cDNA microarray platform used and the raw data of this study are available at the GEO website under the accession numbers GPL1930 and GSE4524, respectively [30]. Slides were scanned on a confocal laser scanner (Arrayexpress; Packard Bioscience, USA) and, for each spot, signal and background intensities were measured using histogram method of Quantarray software (version 3.0, Packard BioScience, BioChip Technologies LLC, USA).

### Statistical Analysis

Data analysis was performed with R project for statistical computing [31] and tools of the associated project, Bioconductor [32]. Prior to analysis, signal intensity was corrected by background subtraction, and data normalized by loess method, using span = 0.4 and degree = 2. For the identification of differentially expressed genes, we used ANOVA model when just one variable was considered. For the identification of differentially expressed genes in a pair-wise manner, we used *t*-test and determined the nominal p-value for each individual gene. Those nominal p-values can be conservatively adjusted for multiple testing with the Bonferroni correction by multiplying them by the number of genes in our chip. For clustering samples on the basis of their expression profile, we applied hierarchical clustering based on correlation distance and complete linkage.

### Northern Blotting

For Northern blot analysis, 15 μg of total RNA was fractionated through a 1% denaturing agarose gel and transferred by capillarity onto Hybond N filters (GE Healthcare BioSciences, NJ, USA). Prehybridization, hybridization, and washes were performed as described by Church and Gilbert [33]. The KLK7 and SOD2 cDNA probes were the same used for immobilization in the array. The human GAPDH cDNA probe was used as control for ensuring equal RNA loading. Probes were labeled by random priming, using Ready-To-Go Labeling Beads (GE Healthcare Bio-Sciences, NJ, USA) and [α-^32^P]dCTP (3000 Ci/mmol). Nylon filters were exposed to Kodak Hyperfilm (GE Healthcare BioSciences, NJ, USA) with intensifying screen.

### Electrophoretic mobility shift assay (EMSA)

Nuclear extracts were obtained from monolayer cultures of PHK, and from cell lines HPV16 and HPV18 treated with 2 nM of human recombinant TNF for 1 h. Briefly, cell plates were washed with ice-cold PBS and cells were scraped in 5 ml of PBS. Cells were transferred to a 15 ml Falcon tube and centrifuged at 3000 rpm for 3 min. Cell pellets were ressuspended in 4 ml of lysis buffer (10 mM HEPES pH 7,9, 10 mM KCl, 0,2 mM EDTA, 1 mM DTT), incubated on ice for 5 min, centrifuged and ressuspended in 4 ml of lysis buffer. Nuclei obtained were centrifuged at 2000 rpm for 2 min, ressuspended in 100 μl of extraction buffer (20 mM HEPES pH7,9, 0,42 M NaCl, 2 mM EDTA, 1 mM DTT, 1 mM PMSF, 2 μM pepstatin, 0,6 μM leupeptin, 25 mU/ml aprotinin) and incubated on ice for 30 min. Finally, the samples were centrifuged at 12000 rpm for 15 min at 4°C. The supernatants were stored at -80°C. The protein concentration was determined by the Bradford method (Bio-Rad, CA, USA).

For gel retardation the following double-stranded oligonucleotide, corresponding to the NF-κB binding sequence, was used: forward-5'-GCCTGGGAAAGTCCCCTCAACT-3' (Invitrogen, CA, USA) was used. The annealed oligonucleotide was labeled with [γ-^32^]ATP (Amersham, Buckinghamshire, UK; 3,000 Ci/mmol) using TK polynucleotide kinase according to the manufacturer instructions (Biolabs, MA, USA) and purified using Sephadex G50 columns followed by phenol:chloroform extraction and precipitation using 10 μg of salmon sperm DNA as a carrier (Invitrogen, CA, USA). DNA pellets were ressuspended in binding buffer (20 mM HEPES pH 7,9, 20% glycerol, 0,1 M KCl, 2 mM EDTA, 1 mM PMSF, 2 μM pepstatin, 0,6 μM leupeptin, 25 mU/ml aprotinin) to a final concentration of 2,5 fmol/μl. The incorporated radioactivity was quantitated using a LS6500 scintillation counter (Beckman Coulter, CA, USA).

The binding of NF-κB was performed in a reaction containing 5 μg of protein extract, 5 μg of BSA, 5 μg of salmon sperm DNA and binding buffer to a final volume of 32 μl on ice. After 10 min, 8 μl of the [γ-32]ATP 5'-end-labeled double-stranded oligonucleotide probe was added, and the incubation was continued for an additional 15 min at 30°C. The DNA-protein complexes were resolved on 4% nondenaturing polyacrylamide gels (29:1 cross-linking ratio), dried, and exposed overnight to X-ray films (Amersham, Buckinghamshire, UK).

## Results

Glass arrays containing 4.800 cDNA sequences were used in order to determine the effects of HPV infection in human keratinocytes as well as the impact of TNF treatment on global gene expression, in HPV negative or positive cells.

**Figure 1:**
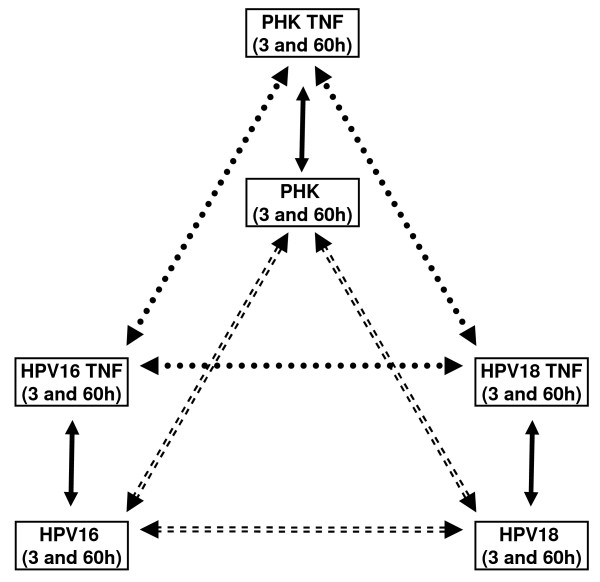
**Experimental setup for the analysis of HPV and TNF effects on keratinocytes gene expression**. In order to characterize and compare the global transcription profile of normal and HPV-immortalized keratinocytes and to analyze their response to TNF we used an experimental setting that allowed us to: 1) identify differential expressed genes between normal PHK, HPV16 and HPV18-immortalized keratinocytes (comparisons represented by dashed arrows); 2) identify genes modulated by TNF upon treatment for three and sixty hours (comparisons represented by solid arrows) and; 3) compare the effect of TNF between normal PHK and cells immortalized by two different high-risk HPV types (comparisons represented by round dot arrows).

In order to identify differentially expressed genes as a function of a unique variable (cell type or TNF-treatment) our dataset was first analyzed by one way ANOVA. The comparisons performed allowed us to determine 1) genes that are differentially expressed between TNF-sensitive and TNF-resistant cells; 2) identify genes that are differentially modulated by TNF at two-time points (3 and 60 hours); 3) analyze the effect of HPV-induced immortalization on TNF-regulated genes and, 4) find genes that are differentially expressed between cells immortalized by two different high-risk HPV types (Figure 1).

### Differentially expressed genes as a function of cell type or TNF treatment

**Figure 2:**
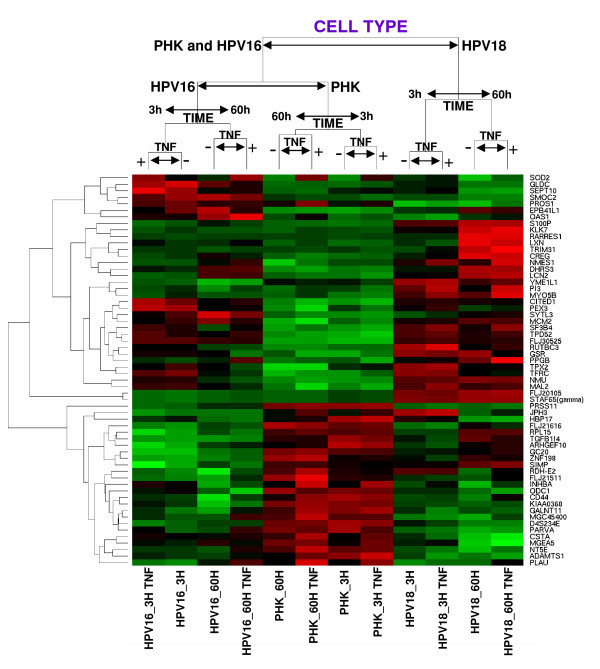
**Hierarchical grouping based on differentially expressed genes as a function of cell type**. These genes where identified by the ANOVA method and the samples where grouped considering the correlation distance and complete linkage. After sample grouping the genes (p values <10^-10^) were hierarchically grouped by their correlation distances. High gene expression is shown in red, low gene expression is shown in green and black indicates non-differential gene expression. Samples: Primary human keratinocytes: controls and treated for 3 or 60 hours with TNF, respectively (PHK_3H, PHK_60H, PHK_3H.TNF, PHK_60H.TNF); HPV16-immortalized keratinocytes: controls and treated for 3 or 60 hours with TNF, respectively (HPV16_3H, HPV16_60H, HPV16_3H.TNF, HPV16_60H.TNF); HPV18-immortalized keratinocytes: controls and treated with 3 or 60 hours for TNF, respectively (HPV18_3H, HPV18_60H, HPV18_3H.TNF, HPV18_60H.TNF).

The identification of differentially expressed genes, with statistical significance, as a function of cell type was performed by ANOVA. Samples and differentially expressed genes (cutoff p-value <10^-10^) were grouped hierarchically, using correlation distance and complete linkage (Figure 2). As it can be observed, normal (PHK) and HPV16-immortalized keratinocytes (HPV16), which are sensitive to TNF cytostatic effect, grouped together while TNF-resistant HPV18-immortalized cell line (HPV18) formed an independent branch. This indicates that TNF-sensitive cell lines share a group of genes which are regulated in a way that clearly differentiate them from the TNF-resistant one. Samples were further clusterized by the time in culture after the last medium change (3 or 60 hs) and finally separated as a function of TNF treatment. This clusterization pattern may reflect differences in cell density and other cultures variables such as nutrients availability or medium conditioning. Initially, all treatments were performed using 30% cell density cultures. As expected, due to TNF cytostatic effect on normal and HPV16-immortalized keratinocytes, cell density at the end of the 60 hours period was different between treated (40–50%) and control cells (70–80%) for these cell lines. On the other hand, both cytokine treated and control HPV18-immortalized cells reached 80–90% cell density by the end of the 60 hours period. Flow-cytometry analysis revealed that the TNF effect on sensitive cells was characterized by the accumulation of cells in the G1-phase of the cell cycle. Conversely, TNF-induced G1-arrest was not observed in HPV18-immortalized keratinocytes [[22,23] and data not shown]. Finally, no differences in cell density were observed for cultures corresponding to 3 hours-treatment group.

**Figure 3:**
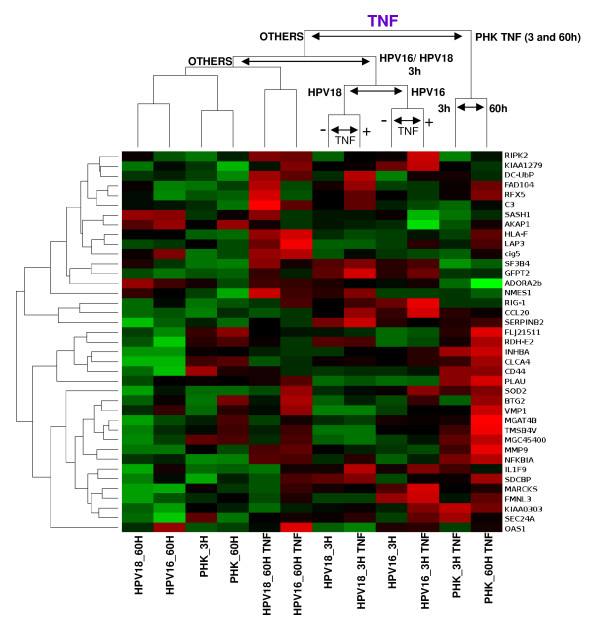
**Hierarchical grouping based on differentially expressed genes as a function of TNF treatment**. These genes where identified by the ANOVA method and the samples where grouped considering the correlation distance and complete linkage. After sample grouping the genes (p values <10^-2,9^) were hierarchically grouped by their correlation distances. High gene expression is shown in red, low gene expression is shown in green and black indicates non-differential gene expression. Samples: Primary human keratinocytes: controls and treated for 3 or 60 hours with TNF, respectively (PHK_3H, PHK_60H, PHK_3H.TNF, PHK_60H.TNF); HPV16-immortalized keratinocytes: controls and treated for 3 or 60 hours with TNF, respectively (HPV16_3H, HPV16_60H, HPV16_3H.TNF, HPV16_60H.TNF); HPV18-immortalized keratinocytes: controls and treated for 3 or 60 hours with TNF, respectively (HPV18_3H, HPV18_60H, HPV18_3H.TNF, HPV18_60H.TNF).

Among the differentially regulated genes we found some related with inflammatory response (*SOD2, TGFB1, CD44, INHBA, OAS1, SIMP*), epidermal development, differentiation and proliferation (*ADAMST1, RARRES1, CREG, HBP17, MCM2, PRSS1, S100P, CREG1*), proteolysis regulation (*KLK7, PI3, LXN*), and cell adhesion (*CD44, PARVA, PROS1*). The name and function of the genes described are listed in Table 1. We next determined the global changes in gene expression as a function of TNF treatment. The name and annotated function of the identified genes that best distinguish samples based on TNF treatment (cutoff p-value <10^-2,9^) are listed in Table 2. As expected, many of these genes are involved in the inflammatory response and/or are direct targets of TNF e.g. CCL20, CD44, HLA-F, IL1F9, NFKBIA, INHBA, SOD2, MARCKS, RFX5. Samples and genes were hierarchically clusterized on the basis of their correlation distance using complete linkage (Figure 3). Samples from TNF-treated normal keratinocytes (PHK) grouped together and apart from the others. HPV-positive samples exhibited a complex clusterization pattern suggesting that the presence of either HPV16 or 18 has an impact on TNF-regulated gene expression. Furthermore, the grouping of treated PHKs apart from the other samples could reflect the fact that PHKs are the only normal cells used in this study and, as such, the only cell type expected to have an unaltered TNF-signaling network. This could contribute to explain the differences in gene expression upon TNF treatment observed between normal and HPV-immortalized keratinocytes.

**Table 1:** Name and function of the differentially expressed genes that best distinguish samples by cell type variable

**GENE**	**UniGene ID**	**GENE NAME**	**FUNCTION**
**ADAMTS1**	Hs.534115	*disintegrin-like and metalloprotease (reprolysin type)*	*negative regulation of cell proliferation*
**ARHGEF10**	Hs.443460	*Rho guanine nucleotide exchange factor (GEF) 10*	*GTPase activator activity*
**CD44**	Hs.502328	*CD44 antigen*	*cell adhesion*
**CITED1**	Hs.40403	*Cbp/p300-interacting transactivator*	*transcription regulator activity*
**CREG**	Hs.5710	*cellular repressor of E1A-stimulated genes 1*	*cell proliferation*
**CSTA**	Hs.518198	*cystatin A (stefin A)*	*cysteine protease inhibitor activity*
**D4S234E**	Hs.518595	*DNA segment on chromosome 4 (unique) 234 expressed sequence*	*dopamine receptor signaling pathway*
**DHRS3**	Hs.289347	*dehydrogenase/reductase (SDR family) member 3*	*fatty acid metabolism*
**EPB41L1**	Hs.437422	*erythrocyte membrane protein band 4.1-like 1*	*structural molecule activity*
**FLJ20105**	Hs.47558	*FLJ20105*	*regulation of transcription*
**FLJ21511**	Hs.479703	*FLJ21511*	*unknown function*
**FLJ21616**	Hs.591836	*FLJ21616*	*regulation of transcription*
**FLJ30525**	Hs.7962	*FLJ30525*	*unknown function*
**GALNT11**	Hs.647109	*UDP-N-acetyl-alpha-D-galactosamine*	*transferase activity, transferring glycosyl groups*
**GC20**	Hs.315230	*translation factor sui1 homolog*	*regulation of translational initiation*
**GLDC**	Hs.584238	*glycine dehydrogenase*	*glycine metabolism*
**GSR**	Hs.271510	*glutathione reductase*	*glutathion metabolism*
**HBP17**	Hs.1690	*fibroblast growth factor binding protein 1*	*regulation of cell proliferation*
**INHBA**	Hs.28792	*inhibin, beta A*	*cell cycle arrest, negative regulation of immune cell differentiation*
**JPH3**	Hs.592068	*junctophilin 3*	*unknown function*
**KIAA0368**	Hs.368255	*KIAA0368*	*ER-associated protein catabolism*
**KLK7**	Hs.151254	*kallikrein 7 (chymotryptic, stratum corneum)*	*epidermis development, proteolysis and peptidolysis, chymotrypsin activity*
**LCN2**	Hs.204238	*lipocalin 2 (oncogene 24p3)*	*transporter activity*
**LXN**	Hs.478067	*latexin*	*enzyme inhibitor activity*
**MAL2**	Hs.201083	*T-cell differentiation protein 2*	*Unknown function*
**MCM2**	Hs.477481	*minichromosome maintenance deficient 2*	*cell cycle*
**MGC45400**	Hs.389734	*transcription elongation factor A (SII)-like 8*	*translation elongation factor activity*
**MGEA5**	Hs.500842	*meningioma expressed antigen 5 (hyaluronidase)*	*glycoprotein catabolism*
**MYO5B**	Hs.200136	*acetyl-Coenzyme A acyltransferase 2*	*fatty acid metabolism*
**NMES1**	Hs.112242	*normal mucosa of esophagus specific 1*	*unknown function*
**NMU**	Hs.418367	*neuromedin U*	*neuropeptide signaling pathway, digestion*
**NT5E**	Hs.153952	*5'-nucleotidase, ecto (CD73)*	*DNA metabolism*
**OAS1**	Hs.524760	*2',5'-oligoadenylate synthetase 1*	*immune response to viral infections*
**ODC1**	Hs.467701	*ornithine decarboxylase 1*	*polyamine biosynthesis*
**PARVA**	Hs.607144	*parvin, alpha*	*cell adhesion, actin binding*
**PEX3**	Hs.7277	*peroxisomal biogenesis factor 3*	*peroxisome organization*
**PI3**	Hs.112341	*protease inhibitor 3, skin-derived (SKALP)*	*elastase-specific inhibitor*
**PLAU**	Hs.77274	*plasminogen activator*	*chemotaxis*
**PPGB**	Hs.517076	*protective protein for beta-galactosidase*	*intracellular protein transport*
**PROS1**	Hs.64016	*protein S (alpha)*	*cell adhesion, endopeptidase inhibitor activity*
**PRSS11**	Hs.501280	*protease, serine, 11 (IGF binding)*	*insulin-like growth facto binding, regulation of cell growth*
**RARRES1**	Hs.131269	*retinoic acid receptor responder*	*negative regulation of cell proliferation*
**RDH-E2**	Hs.170673	*epidermal retinal dehydrogenase 2*	*oxidoreductase activity*
**RPL15**	Hs.381219	*ribosomal protein L15*	*protein biosynthesis*
**RUTBC3**	Hs.474914	*RUN and TBC1 domain containing 3*	*unknown function*
**S100P**	Hs.440880	*S100 calcium binding protein P*	*cell cycle progression and differentiation*
**SEPT10**	Hs.469615	*septin 10*	*cell cycle*
**SF3B4**	Hs.516160	*myotubularin related protein 11*	*inositol or phosphatidylinositol phosphatase activity*
**SIMP**	Hs.475812	*immunodominant MHC-associated peptides*	*protein amino acid glycosylation*
**SMOC2**	Hs.487200	*SPARC related modular calcium binding*	*calcium ion binding*
**SOD2**	Hs.487046	*superoxide dismutase 2*	*age-dependent response to reactive oxygen species, cellular defense response*
**STAF65 (gamma)**	Hs.6232	*SPTF-associated factor 65 gamma*	*regulation of transcription, DNA- dependent*
**SYTL3**	Hs.436977	*synaptotagmin-like 3*	*intracellular protein transport*
**TFRC**	Hs.529618	*transferrin receptor (p90, CD71)*	*endocytosis*
**TGFB1**	Hs.645227	*transforming growth factor, beta 1*	*cell proloferation*
**TPD52**	Hs.368433	*tumor protein D52*	*morphogenesis*
**TPX2**	Hs.244580	*microtubule-associated protein homolog*	*cell proliferation*
**TRIM31**	Hs.493275	*tripartite motif-containing 31*	*protein ubiquitination, ubiquitin ligase activity*
**YME1L1**	Hs.499145	*YME1-like 1 (S. cerevisiae)*	*protein catabolism*
**ZNF198**	Hs.644041	*zinc finger protein 198*	*regulation of transcription, DNA- dependent*

*Genes are listed in alphabetical order. The cutoff p-value was set as <10^-10^.

**Table 2:** Name and function of the differentially expressed genes that best distinguish samples by TNF treatment variable

**GENE**	**UniGene ID**	**GENE NAME**	**FUNCTION**
**ADORA2b**	Hs.167046	*adenosine A2b receptor*	*activation of MAPK*
**AKAP1**	Hs.463506	*A kinase (PRKA) anchor protein 1*	*RNA binding*
**BTG2**	Hs.519162	*BTG family, member 2*	*negative regulation of cell proliferation*
**C3**	Hs.529053	*complement component 3*	*inflammatory response*
**CCL20**	Hs.75498	*chemokine (C-C motif) ligand 20*	*inflammatory response*
**CD44**	Hs.502328	*CD44 antigen*	*cell adhesion*
**cig5**	Hs.17518	*radical S-adenosyl methionine domain containing 2*	*Catalytic activity*
**CLCA4**	Hs.546343	*chloride channel, calcium activated, family member 4*	*chloride transport*
**DC-UbP**	Hs.179852	*dendritic cell-derived ubiquitin-like protein*	*Protein modification*
**FAD104**	Hs.159430	*fibronectin type III domain containing 3B*	*cell differentiation*
**FLJ21511**	Hs.479703	*FLJ21511*	*Unknown function*
**FMNL3**	Hs.179838	*formin-like 3*	*cell organization and biogenesis*
**GFPT2**	Hs.30332	*glutamine-fructose-6-phosphate transaminase 2*	*carbohydrate biosynthesis*
**HLA-F**	Hs.519972	*major histocompatibility complex, class I, F*	*antigen presentation, endogenous antigen*
**IL1F9**	Hs.211238	*interleukin 1 family, member 9*	*inflammatory response*
**INHBA**	Hs.28792	*inhibin, beta A*	*cell cycle arrest, negative regulation of immune cell differentiation*
**KIAA0303**	Hs.133539	*microtubule associated serine/threonine kinase family member 4*	*protein kinase activity*
**KIAA1279**	Hs.279580	*KIAA1279*	*Unknown function*
**LAP3**	Hs.479264	*leucine aminopeptidase 3*	*Protein metabolism*
**MARCKS**	Hs.75061	*MARCKS-like 1*	*calmodulin binding, macrophage activation*
**MGAT4B**	Hs.437277	*mannosyl (alpha-1,3-)-glycoprotein beta-1,4-N-acetylglucosaminyltransferase, isoenzyme B*	*cytokine activity*
**MGC45400**	Hs.389734	*transcription elongation factor A (SII)-like 8*	*translation elongation factor activity*
**MMP9**	Hs.297413	*matrix metalloproteinase 9*	*proteolysis and peptidolysis*
**NFKBIA**	Hs.81328	*nuclear factor of kappa light polypeptide gene enhancer in B-cells inhibitor, alpha*	*cytoplasmic sequestering of NF-kappaB*
**NMES1**	Hs.112242	*normal mucosa of esophagus specific 1*	*Unknown function*
**OAS1**	Hs.524760	*2',5'-oligoadenylate synthetase 1*	*immune response to viral infections*
**PLAU**	Hs.77274	*plasminogen activator*	*chemotaxis*
**RDH-E2**	Hs.170673	*epidermal retinal dehydrogenase 2*	*oxidoreductase activity*
**RFX5**	Hs.166891	*regulatory factor X, 5*	*inflammatory response, HLA class II expression*
**RIG-1**	Hs.17466	*retinoic acid receptor responder (tazarotene induced) 3*	*negative regulation of cell proliferation*
**RIPK2**	Hs.103755	*receptor-interacting serine-threonine kinase 2*	*inflammatory response*
**SASH1**	Hs.193133	*SAM and SH3 domain containing 1*	*Negative regulation of cell cycle*
**SDCBP**	Hs.200804	*syndecan binding protein (syntenin)*	*intracellular signaling cascade, interleukin-5 receptor binding*
**SEC24A**	Hs.211612	*SEC24 related gene family, member A*	*intracellular protein transport*
**SERPINB2**	Hs.514913	*encoding serine (or cysteine) proteinase inhibitor, clade B (ovalbumin), member 2*	*anti-apoptosis*
**SF3B4**	Hs.412818	*myotubularin related protein 11*	*RNA splicing*
**SOD2**	Hs.487046	*superoxide dismutase 2*	*age-dependent response to reactive oxygen species, cellular defense response*
**TMSB4**	Hs.522584	*thymosin, beta 4, X-linked*	*cytoskeleton organization and biogenesis*
**VMP1**	Hs.444569	*transmembrane protein 49*	*Unknown function*

*Genes are listed in alphabetical order. The cutoff p-value was set as <10^-2,9^.

Since our experimental setting included the comparative analysis of global gene expression at two time points (3 or 60 hours), we searched for genes that best differentiate our samples as a function of time. We found 48 genes that clearly differentiate samples from the analyzed time points. The name and annotated function of the identified genes (cutoff p-value <10^-9^) are listed in additional file 1. Hierarchical clusterization divided samples in two main branches (additional file 2). Each branch was exclusively composed of samples from the same time point, namely, 3 or 60 hours. Samples from the 3 hours-time point formed a secondary branch that divided normal from HPV-immortalized keratinocytes. On the other hand, samples from the 60 hours-time point formed a secondary branch that divided normal and HPV16-immortalized keratinocytes (TNF-sensitive samples) from HPV18-immortalized keratinocytes (TNF-resistant samples).

### Differentially expressed genes between TNF-sensitive and TNF-resistant cells

**Figure 4:**
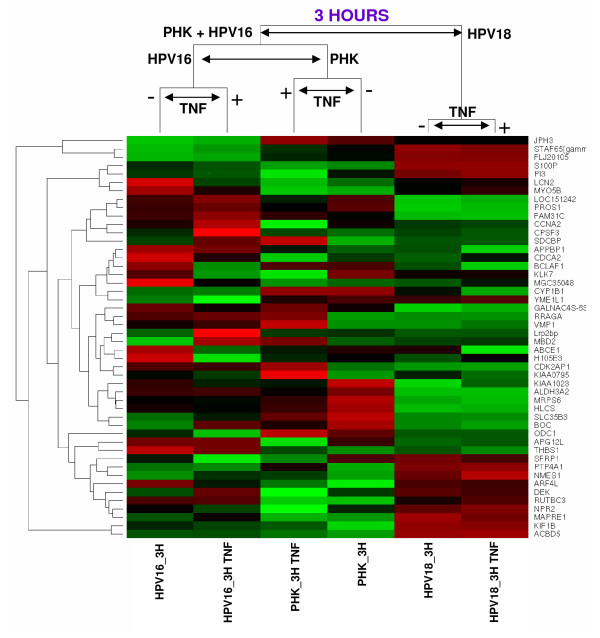
**Supervised hierarchical grouping based on differentially expressed genes between normal/HPV16-immortalized keratinocytes and HPV 18-immortalized ones after treatment with TNF for 3 hours**. High gene expression is shown in red, low gene expression is shown in green and black indicates non-differential gene expression. Samples: Primary human keratinocytes: controls and treated for 3 hours with TNF, respectively (PHK_3H, PHK_3H.TNF); HPV16-immortalized keratinocytes: controls and treated for 3 hours with TNF, respectively (HPV16_3H, HPV16_3H.TNF); HPV18-immortalized keratinocytes: controls and treated for 3 hours with TNF, respectively (HPV18_3H, HPV18_3H.TNF).

In order to identify differentially expressed genes between specific samples we performed a series of pair-wise comparisons. For each pair-wise comparison, we generated a list of differentially expressed genes with p-value lower than 0,01. The complete list of all pair-wise comparisons performed is presented in additional file 3. We next aimed to characterize genes that were differentially expressed between TNF-sensitive (PHK and HPV16) and TNF-resistant cells (HPV18). To achieve this goal we selected the thirty genes with the lowest p-values that best distinguish both PHK and HPV16 from HPV18 and the thirty genes with lowest p-values that best distinguish both PHK+TNF and HPV16+TNF from HPV18+TNF (considering treatment with TNF for 3 h). Twelve genes were common to both lists giving a total of 48 different genes identified (Table 3). Using the expression profile of these 48 genes, samples were grouped hierarchically, based on their correlation distance and complete linkage (Figure 4).

**Table 3:** List of differentially expressed genes that best distinguish TNF-resistant cells (HPV18) from TNF-sensitive cells (PHK and HPV16), in normal culture conditions or upon treatment with TNF for 3 hours

		**(PHK and HPV16) *vs *HPV18**		**(PHK_TNF and HPV16_TNF) *vs *HPV18_TNF**	
			
**GENE**	**GENE NAME**	**FOLD**	**p VALUE**	**FOLD**	**p VALUE**
**ABCE1**	*ATP-binding cassette, sub-family E (OABP), member 1*	0.592	0.001384	----	----
**ACBD5**	*acyl-Coenzyme A binding domain containing 5*	----	----	0.378	0.00086
**ALDH3A2**	*encoding aldehyde dehydrogenase 3 family, member A2*	1.623	0.00122	----	----
**APG12L**	*APG12 autophagy 12-like (S. cerevisiae)*	1.739	0.000924	----	----
**APPBP1**	*amyloid beta precursor protein binding protein 1*	0.540	0.000283	----	----
**ARF4L**	*ADP-ribosylation factor 4-like*	----	----	0.578	5.70E-05
**BCLAF1**	*BCL2-associated transcription factor 1*	0.611	0.000876	----	----
**BOC**	*brother of CDO*	----	----	2.337	6.00E-06
**CCNA2**	*cyclin A2*	0.577	0.000604	----	----
**CDCA2**	*cell division cycle associated 2*	----	----	0.539	7.20E-05
**CDK2AP1**	*CDK2-associated protein 1*	----	----	1.523	0.000148
**CPSF3**	*cleavage and polyadenylation specific factor 3*	0.586	0.000466	----	----
**CYP1B1**	*cytochrome P450, family 1, subfamily B, polypeptide 1*	0.499	0.001386	----	----
**DEK**	*DEK oncogene*	0.480	0.000278	----	----
**FAM31C**	*family with sequence similarity 31, member C*	----	----	2.548	0.000663
**FLJ20105**	*hypothetical protein LOC54821*	0.026	5.00E-06	0.029	2.00E-06
**GALNAC4S-6ST**	*B cell RAG associated protein*	1.827	0.000145	1.673	0.000215
**H105E3**	*encoding NAD(P) dependent steroid dehydrogenase-like*	----	----	0.569	6.80E-05
**HLCS**	*holocarboxylase synthetase*	----	----	1.551	0.00011
**JPH3**	*junctophilin 3*	0.154	0.000192	----	----
**KIAA0795**	*kelch-like 18 (Drosophila)*	0.857	0.001325	----	----
**KIAA1023**	*IQ motif containing E*	1.575	0.000723	----	----
**KIF1B**	*kinesin family member 1B*	----	----	0.570	0.000632
**KLK7**	*encoding kallikrein 7 (chymotryptic, stratum corneum)*	0.421	0.000416	0.374	2.30E-05
**LCN2**	*lipocalin 2 (oncogene 24p3)*	----	----	0.216	0.000686
**LOC151242**	*protein phosphatase 1, regulatory (inhibitor)*	1.928	0.000268	----	----
**Lrp2bp**	*low density lipoprotein receptor-related protein binding protein*	0.629	0.000574	----	----
**MAPRE1**	*encoding microtubule-associated protein, RP/EB family, member 1*	0.410	0.001068	0.503	4.20E-05
**MBD2**	*methyl-CpG binding domain protein 2*	0.680	0.001247	----	----
**MGC35048**	*hypothetical protein MGC35048*	----	----	0.499	0.000211
**MRPS6**	*mitochondrial ribosomal protein S6*	----	----	1.460	0.000161
**MYO5B**	*acetyl-Coenzyme A acyltransferase 2*	0.353	0.000104	0.292	4.10E-05
**NMES1**	*normal mucosa of esophagus specific 1*	0.324	0.000294	0.244	7.00E-06
**NPR2**	*encoding natriuretic peptide receptor B/guanylate cyclase B*	----	----	0.435	0.000234
**ODC1**	*ornithine decarboxylase 1*	1.660	0.000886	----	----
**PI3**	*protease inhibitor 3, skin-derived (SKALP)*	0.213	0.000274	0.207	1.80E-05
**PROS1**	*protein S (alpha)*	1.807	0.000633	----	----
**PTP4A1**	*protein tyrosine phosphatase type IVA, member 1*	0.478	0.000385	0.497	1.80E-05
**RRAGA**	*Ras-related GTP binding A*	----	----	1.510	0.000301
**RUTBC3**	*RUN and TBC1 domain 3*	0.518	0.000704	0.450	0.000131
**S100P**	*S100 calcium binding protein P*	0.101	0	0.102	0
**SDCBP**	*syndecan binding protein (syntenin)*	0.456	0.000395	----	----
**SFRP1**	*secreted frizzled-related protein 1*	----	----	0.502	5.10E-05
**SLC35B3**	*solute carrier family 35, member B3*	----	----	1.990	0.000304
**STAF65 (gamma)**	*SPTF-associated factor 65 gamma*	0.021	1.00E-06	0.028	0
**THBS1**	*thrombospondin 1*	----	----	2.712	0.000225
**VMP1**	*likely ortholog of rat vacuole membrane protein 1*	----	----	1.547	0.000899
**YME1L1**	*YME1-like 1 (S. cerevisiae)*	0.373	2.60E-05	0.333	5.00E-06

*Genes are listed in alphabetical order. Underlined genes were identified as differentially expressed between TNF sensitive and TNF resistant cells in both culture conditions.

Using this approach we observed that genes involved with cell cycle control (CCNA2, CDCA2, CDK2AP1), epidermis development, differentiation and proliferation (KLK7, ALDH3A2, PI3, APG12L, BCLAF1, DEK, MAPRE1, S100P, RRAGA, SFRP1), protein ubiquitination (APPBP1) and cell adhesion (BOC, PROS1, SDCBP, THBS1, JPH3), among others, were differentially expressed between TNF-sensitive and TNF-resistant cells (Table 3). These analyses were also performed considering TNF treatment for 60h (available as additional files 4 and 5).

### Validation on KLK7 and SOD2 as differentially expressed genes

**Figure 5:**
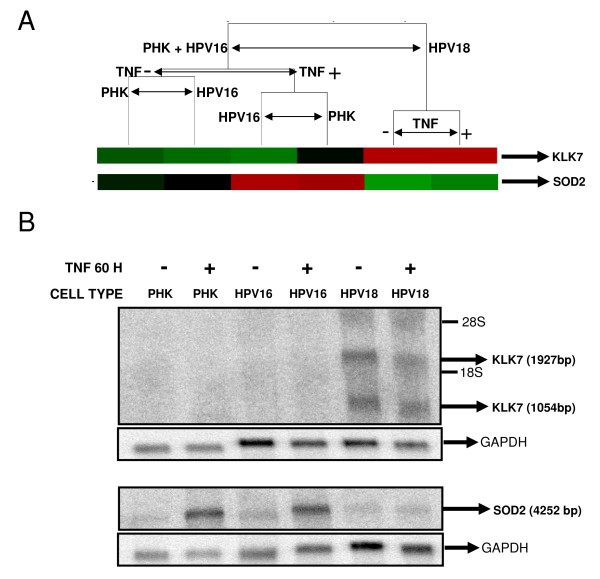
**Differential expression of KLK7 and SOD2 transcripts**. **A**. Detail of the supervised hierarchical grouping based on differentially expressed genes between normal/HPV16-immortalized keratinocytes and HPV 18-immortalized ones, after treatment with TNF for 60 hours. **B**. Northern blot analysis of KLK7 and SOD2 transcription levels. Arrows indicate the two alternative splicing products of KLK7 in HPV18-immortalized keratinocytes (*GenBank *# NM_005046); the SOD2 transcript is induced by TNF in both PHK and HPV16-immortalized cells but not in HPV18-immortalized cells (*GenBank *# NM_00636). A probe against GAPDH was used to monitor comparable loading between samples.

We identified a group of genes whose differential expression could be associated with the differential response to TNF of the cell lines studied, namely: KLK7 (kallikrein 7), SOD2 (*superoxide dismutase 2*), S100P (*S100 calcium binding protein P*), PI3 (*protease inhibitor 3, skin-derived*), CSTA (*cystatin A*), RARRES1 (*retinoic acid receptor responder 1*), and LXN (*latexin*). Based on the reported function as well as the expression profile observed, KLK7 and SOD2 genes were selected for further analysis. The expression pattern of these genes observed by microarray was confirmed by Northern Blot in control and TNF-treated (60 hours) samples from all cell lines used (Figures 5A and 5B). As it can be observed, KLK7 is equally expressed in TNF-treated or untreated HPV18-immortalized cells but is not detected in PHK or HPV16-immortalized cells, even after cytokine treatment. On the other hand, we observed that SOD2 expression is up-regulated by TNF in both PHK and HPV16-immortalized cells but not in HPV18-immortalized cells, confirming the data obtained by microarray (Figures 5A and 5B).

### NF-κB is differentially activated in HPV-16- and HPV-18-infected cells

**Figure 6:**
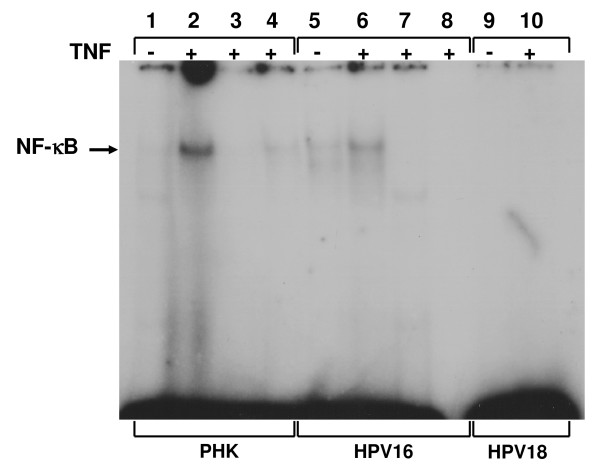
**Analysis of TNF-induced NF-κB activation in normal and HPV16 or 18-immortalized keratinocytes**. Subconfluent cultures of normal and HPV16 or 18-immortalized keratinocytes treated with 2 nM of TNF for 1 h were used to obtain nuclear protein extracts. For each EMSA reaction, 5 μg of nuclear protein were incubated with 50 fmol of [γ-32P]ATP-labeled double-stranded oligonucleotide and a 50X excess of unlabeled oligonucleotide (lanes 3 and 7). Specificity of binding was further demonstrated by incubation of 1 μg of nuclear protein with the described amount of labeled consensus oligonucleotide and a 50X excess of a labeled oligonucleotide carrying a single-base mutation at the NF-κB binding site (lane 4), and incubation of nuclear extract in the absence of any labeled probe (lane 8). NF-κB DNA binding reactions were carried out as described under "Material and Methods". DNA binding complexes are indicated.

It has been reported that NF-κB activation plays an important role in SOD2 induction by TNF. So we hypothesized that the differential expression of SOD2 could be due to the presence of different levels of activated NF-κB after TNF treatment between TNF-sensitive and TNF resistant cells. In order to address this hypothesis NF-κB activation was determined by electrophoretic mobility shift assay (EMSA) using specific oligonucleotides and nuclear protein extracts. Interestingly, we observed that normal as well as HPV16-immortalized keratinocytes exhibited a clear activation of NF-κB as shown by the increase of this factor levels in nuclear protein extracts after TNF treatment (Figure 6). On the other hand, NF-κB activation in TNF-resistant HPV18-immortalized cells was below the level of detection (Figure 6, lanes 9 and 10). This prompted us to analyze if NF-κB activation was also altered in other HPV-positive cell lines previously reported to be resistant to TNF cytostatic effect [[22,23], and data not shown]. To address this issue we performed EMSA using nuclear protein extracts obtained from HPV16-positive (SiHa) or HPV18-positive (HeLa and SW756) cervical cancer derived cell lines cultures. We observed that TNF-resistant cells exhibited reduced NF-κB activation when compared to normal PHK (additional file 6). Altogether, these observations suggest that alteration of TNF-signaling pathway leading to NF-κB activation is a common event in HPV-positive cell lines resistant to this cytokine.

## Discussion

Production and secretion of inflammatory cytokines are among the main events that take place upon viral infection. These molecules coordinate host cell-mediated immune response by recruiting cellular elements from the immune system and by regulating gene expression on target cells [34,35]. The pleiotropic cytokine TNF is a key regulator of inflammation of the epithelia with a well-documented capacity to induce growth arrest in normal or HPV16-immortalized keratinocytes, mainly in the G0/G1 phase of the cell cycle [36]. Conversely, we have previously reported that HPV18-immortalized and both HPV16 or HPV18-transformed cell lines are resistant to TNF-induced growth arrest [22,23].

In order to address the yet unknown molecular bases of this difference we applied cDNA microarray technology to compare the global gene expression profiles of TNF-sensitive normal and HPV16-immortalized keratinocytes with that of TNF-resistant HPV18-immortalized ones. Some limitations of this study are the use of a reduced number of samples and the existence of differences in cell culture conditions which are inherent to our experimental setting, i.e. the differences in cell density at the different time-points described above. However, using this approach we identified a group of genes that clearly distinguish both cells groups (Figure 2 and Table 1). This indicates that TNF-sensitive cell lines share a group of genes which are regulated in a way that clearly differentiate them from the TNF-resistant one.

On the other hand, when we analyzed changes in global gene expression as a function of TNF treatment we observed that HPV16 and HPV18 samples could not be distinguished from each other while normal keratinocytes could be readily discriminated (Figure 3). This observation suggests that the presence of either HPV16 or 18 has an impact on TNF-regulated gene expression. In line with these observations, several studies have shown that HPV positive cells exhibit impaired TNF pathways [37,38]. Moreover, it has been reported that the effects of TNF on HPV-harboring cells depends on variables as cell type studied, the virus type present and culture conditions (i.e., growth factors availability). This cytokine is capable of inducing the proliferation of HPV16-immortalized human cervical epithelial cells cultures in the absence of growth factors through an autocrine, EGF receptor-dependent, pathway [39]. Besides, TNF can upregulate E6/E7 RNA expression and cyclin-dependent kinase activity in these cells [40]. Conversely, it has been reported that TNF exerts a potent cytostatic effect on HPV16-immortalized keratinocytes while HPV18-immortalized as well as cervical carcinoma-derived HPV-positive cell lines remain unaffected [22,23]. Furthermore, it has been observed that increased tumorigenicity of human keratinocytes transformed by HPV16 is associated with resistance to TNF cytostatic effect [24]. Finally, it was demonstrated that TNF downregulates HPV18 transcription in non-malignant HeLa-fibroblasts hybrids, while viral expression in tumorigenic hybrids segregants as well as in parental HeLa cells remained undisturbed [25]. On the other hand, it has been consistently observed that TNF negatively regulates normal keratinocytes proliferation in monolayer [22,23,36] as well as in organotypic cell cultures [41,42]. Altogether, these data support the notion that acquisition of resistance to TNF by HPV-infected cells may represent an important step towards malignancy.

Despite the existence of similarities between the two high-risk HPV types used to generate the cell lines studied, the fact that HPV16 and HPV18 are different viruses that exhibit clear differences in their biological activities must be highlighted. For instance, epidemiological studies have shown that HPV18 is more associated to cervical adenocarcinomas while HPV16 is more prevalent in squamous cell carcinomas [43-45]. Furthermore, compared to other HPV types HPV18 has been associated with increased transforming potential in cell culture systems and with poorer cancer prognosis at the clinical level [26,27,46,47]. On the other hand, HPV16 exhibits a greater potential to establish persistent infections that can progress to high-grade lesions [48,49]. Although we cannot explain the molecular bases of the differences in gene expression between these cell lines, we believe that this may reflect the divergences that exist between these HPV types.

We next searched for genes that best distinguish between TNF-sensitive and TNF-resistant cells by pair-wise comparison both before and after cytokine treatment for 3 or 60 hours. By this means we identified 48 and 52 different genes, respectively, that set apart TNF-sensitive from TNF resistant cells (Figure 4, Table 3, additional files 4 and 5). The functional characterization of these genes shows that they are involved in critical cellular processes such as regulation of proliferation, differentiation and cell adhesion. Altogether, the differential expression of these genes may contribute to the differential response to the cytostatic effect of TNF observed in these cells.

Two genes, namely KLK7 and SOD2, were selected for further analysis based on their reported function and expression profile (Figure 5A). KLK7 expression pattern was validated by Northern blot and showed that it is equally expressed in TNF-treated or untreated HPV18-immortalized cells but is not detected in PHK or HPV16-immortalized cells (Figure 5B). Kallikreins are a sub-group of serine proteases with different physiological functions. In humans, kallikreins are encoded by 15 structurally similar, steroid hormone-regulated genes that co-localize to chromosome 19q13.4, representing the largest cluster of contiguous protease genes in the entire genome [50-52]. These proteins mediate the proteolytic degradation of cohesive intracellular structures associated to epithelial differentiation. Recent data also suggest that kallikreins may be causally involved in carcinogenesis, particularly in tumor metastasis and invasion, and, thus, may represent attractive drug targets to consider for therapeutic intervention [50]. Consistent with our findings, it has been observed that KLK7 expression is up-regulated in cervical tumors as well as in cells lines derived from them. On the other hand, normal keratinocytes express low levels of this protein [53,54]. Furthermore, KLK7 expression has been found up-regulated in breast [55] and ovary tumors [56] and is being considered a new tumor progression marker.

The superoxide dismutase 2 (SOD2) expression pattern was also validated by Northern blot (Figure 5B). This gene is up-regulated in TNF-sensitive but not in TNF-resistant cells. The superoxide dismutase 2 (SOD2) belongs to a family of enzymes involved in the conversion of superoxide radicals in molecular oxygen. Reactive oxygen metabolites have multifactorial effects on the regulation of cell growth and malignant invasion. Furthermore, numerous in vivo studies have shown that the superoxide dismutases can be highly expressed in aggressive human solid tumors [57-59].

Previous reports have shown that activation of the transcription factor NF-κB is essential for the induction of SOD2 by TNF and IL-1β [60,61]. Here we show that TNF-sensitive cells exhibit higher levels of activated NF-κB than TNF-resistant ones after cytokine treatment (Figure 6 and additional file 6). Several studies have shown that NF-κB is a negative regulator of keratinocytes proliferation in the epidermis, and that it plays an important role in cell differentiation and tissue homeostasis [62-64]. In stratified epithelia NF-κB is found in the cytoplasm of proliferating cells from the basal layer while it is detected in the nuclei of non-proliferating cells from the upper layers. Furthermore, it has been observed that NF-κB superexpression is associated with epidermal hypoplasia while its down-regulation promotes hyperplasia [62]. Overall, these data suggest that alterations in TNF-mediated NF-κB activation pathways can play a role in the development and progression of HPV-associated epithelial and mucosal lesions.

## Conclusion

Progression of HPV-associated lesions depends on the many alterations caused by this virus in the infected cells. We have identified multiple genes differentially regulated by TNF in HPV16 and HPV18 immortalized keratinocytes. Among them we found KLK7 (*kallikrein 7*), SOD2 (*superoxide dismutase 2*), S100P (*S100 calcium binding protein P*), PI3 (*protease inhibitor 3, skin-derived*), CSTA (*cystatin A*), RARRES1 (*retinoic acid receptor responder 1*), and LXN (*latexin*). The differential expression of the *KLK7 *and *SOD2 *transcripts was further confirmed at the RNA level. Moreover, we present evidence that differential *SOD2 *expression correlates with the levels of NF-κB activation exhibited by TNF-sensitive and TNF-resistant cells.

This is the first time that the effect of TNF on global gene expression of normal and HPV-immortalized keratinocytes is addressed at two time points. The thorough analysis of the expression pattern of the identified genes may contribute to the understanding of critical differences between transient and chronic events. Furthermore, it may provide insights of the molecular mechanisms of HPV-induced TNF resistance, contribute to the identification of key functions and pathways associated to specific HPV types and, finally, lead to the identification of new cervical tumor progression markers.

## Competing interests

The authors declare that they have no competing interests.

## Authors' contributions

LT conceived the study, participated in its design, was involved in cell culture, microarray studies, gene expression validation and manuscript preparation. EB was involved in co-ordination, EMSA assays and manuscript preparation. GHE, RHJ and EJN were involved in microarray data analyses and statistical modeling. WKM and AELC were involved in RNA amplification and microarray studies. LLV participated in the study design and co-ordination. LFLR conceptualized the study format. All authors have read and approved the final version of the manuscript.

## Pre-publication history

The pre-publication history for this paper can be accessed here:



## Supplementary Material

Additional file 1 Table with name and function of the differentially expressed genes that best distinguish samples by time variable. The cutoff p-value was set as <10^-9^. Click here for file

Additional file 2 Hierarchical grouping based on differentially expressed genes as a function of time. These genes where identified by the ANOVA method and the samples where grouped considering the correlation distance and complete linkage. After sample grouping the genes were hierarchically grouped by their correlation distances. High gene expression is shown in red, low gene expression is shown in green and black indicates non-differential gene expression, p values <10^-9^. Samples: Primary human keratinocytes: controls and treated for 3 or 60 hours with TNF, respectively (PHK_3H, PHK_60H, PHK_3H.TNF, PHK_60H.TNF); HPV16-immortalized keratinocytes: controls and treated for 3 or 60 hours with TNF, respectively (HPV16_3H, HPV16_60H, HPV16_3H.TNF, HPV16_60H.TNF); HPV18-immortalized keratinocytes: controls and treated for 3 or 60 hours with TNF, respectively (HPV18_3H, HPV18_60H, HPV18_3H.TNF, HPV18_60H.TNF). Click here for file

Additional file 3 Table of differentially expressed genes obtained by pair-wise comparison of all the samples and their respective expression ratio ordered by p-value. PHK_3H (primary human keratinocytes – control/3 hours), PHK_60H (primary human keratinocytes – control/60 hours), HPV16_3H (HPV16-immortalized keratinocytes – control/3 hours), HPV16_60H (HPV16-immortalized keratinocytes – control/60 hours), HPV18_3H (HPV18-immortalized keratinocytes – control/3 hours), HPV18_60H (HPV18-immortalized keratinocytes – control/60 hours), PHK_TNF3H (primary human keratinocytes – TNF/3 hours), PHK_TNF60H (primary human keratinocytes – TNF/60 hours), HPV16_TNF3H (HPV16-immortalized keratinocytes – TNF/3 hours), HPV16_TNF60H (HPV16-immortalized keratinocytes – TNF/60 hours), HPV18_TNF3H (HPV18-immortalized keratinocytes – TNF/3 hours), HPV18_TNF60H (HPV18-immortalized keratinocytes – TNF/60 hours). Click here for file

Additional file 4 Supervised hierarchical grouping based on the 30 genes that best differentiate normal and HPV 16-immortalized keratinocytes from the HPV 18-immortalized ones after treatment with TNF for 60 hours. High gene expression is shown in red, low gene expression is shown in red and black indicates non-differential gene expression. PHK_3H (primary human keratinocytes – control/3 hours), PHK_3H TNF (primary human keratinocytes – TNF/3 hours), HPV16_3H (HPV16-immortalized keratinocytes – control/3 hours), HPV16_3H TNF (HPV16-immortalized keratinocytes – TNF/3 hours), HPV18_3H (HPV18-immortalized keratinocytes – control/3 hours), HPV18_3H TNF (HPV18-immortalized keratinocytes – TNF/3 hours). Click here for file

Additional file 5 Table of differentially expressed genes that best distinguish TNF-resistant (HPV18) from TNF-sensitive cells (PHK and HPV16), in normal culture conditions or upon treatment with TNF for 60 hours. Click here for file

Additional file 6 Analysis of TNF-induced NF-κB activation in normal and HPV16 or 18-transformed keratinocytes. Nuclear protein extracts were obtained from sub confluent cultures of normal keratinocytes (PHK), HPV16-positive (SiHa) and HPV18-positive (SW756 and HeLa) cervical cancer-derived cell lines treated with 2 nM of TNF for 1 h. For each EMSA reaction, 5 mg of nuclear protein were incubated with 50 fmol of [γ-32P]ATP-labeled double-stranded oligonucleotide and a 50X excess of consensus unlabeled oligonucleotide (lanes 4, 8 and 13). Specificity of binding was further demonstrated by incubation of nuclear extracts with the described amount of labeled consensus oligonucleotide and a 50X excess of a labeled oligonucleotide carrying a single-base mutation at the NF-κB binding site (lanes 3, 7 and 14) and incubation of nuclear extract in the absence of any labeled probe (lane 15). NF-κB-DNA binding reactions were carried out as described under "Material and Methods". HeLa nuclear extracts obtained from two different experiments were included as controls of NF-κB activation levels in HPV-positive cell lines. DNA binding complexes are indicated. Click here for file
