# Supplementary material for: Characterization of global transcription profile of normal and HPV-immortalized keratinocytes and their response to TNF treatment
Source: BMC Med Genomics. 2008 Jun 27;1:29. doi: 10.1186/1755-8794-1-29 (PMC2459201; doi:10.1186/1755-8794-1-29)
Supplement: Additional file 1 — Table with name and function of the differentially expressed genes that best distinguish samples by time variable. The cutoff p-value was set as <10-9. [file 1755-8794-1-29-S1.pdf]

GENE	UniGene ID	GENE NAME	FUNCTION
**BCL2L13**	*Hs.699302*	*BCL2-like 13*	*apoptosis facilitator*
**C20orf1**	---------	---------	---------
**CARS**	*Hs.274873*	*cysteinyl-tRNA synthetase*	*Aminoacyl-tRNA biosynthesis, Cysteine metabolism*
**CAV1**	*Hs.74034*	*caveolin 1*	*cholesterol homeostasis, cholesterol transport, inactivation of MAPK activity*
**CCL20**	*Hs.75498*	*chemokine (C-C motif) ligand 20*	*inflammatory response*
**COL4A1**	*Hs.17441*	*collagen, type IV, alpha 1*	*extracellular matrix structural constituent*
**DHX9/DDX9**	*Hs.191518*	*DEAH (Asp-Glu-Ala-His) box polypeptide 9*	*ATP binding*
**DKFZP564F0522**	*Hs.468140*	*DKFZP564F0522*	*phosphopantetheine binding*
**DKFZp761H0421**	*Hs.632255*	*RUN domain containing 1*	*unknown function*
**ELF3**	*Hs.67928*	*E74-like factor 3 (ets domain transcription factor, epithelial-specific)*	*epidermis development*
**FLJ12787**	*Hs.406703*	*Src-associated protein SAW*	*cell cycle control and apoptosis*
**FLJ21870**	*Hs.355455*	*ANKRD57*	
**FLJ22087**	*Hs.591183*	*amplified in breast cancer 1*	*unknown function*
**FLJ23476**	*Hs.301564*	*yrdC domain containing*	*negative regulation of transport*
**FLJ25804**	*Hs.33366*	*cell division cycle associated 2*	*unknown function*
**GFPT2**	*Hs.30332*	*glutamine-fructose-6-phosphate transaminase 2*	*carbohydrate biosynthesis*
**GPR56**	*Hs.513633*	*G protein-coupled receptor 56*	*cell adhesion*
**HBP17**	*Hs.1690*	*fibroblast growth factor binding protein 1*	*regulation of cell proliferation*
**HMGE**	*Hs.443723*	*GrpE-like 1*	*unfolded protein binding, adenyl-nucleotide exchange factor activity*
**Hs.S2140567**	*Hs.485233*	*mitogen-activated protein kinase kinase kinase 14*	*Apoptosis, Keratinocyte Differentiation, MAPK signaling pathway, NF-kB Signaling Pathway*
**HSPH1**	*Hs.36927*	*heat shock 105kDa/110kDa protein 1*	*protein folding*
**KIAA0063**	*Hs.3094*	*Josephin domain containing 1*	*unknown function*
**KIAA0247**	*Hs.440025*	*KIAA0247*	*unknown function*
**KIAA0438**	*Hs.483036*	*praja 2*	*ligase activity*
**KLK7**	*Hs.151254*	*kallikrein 7 (chymotryptic, stratum corneum)*	*epidermis development, proteolysis and peptidolysis, chymotrypsin activity*
**LOC112970**	*Hs.476033*	*thioredoxin domain containing 12*	*Glutathione metabolism*
**LSM3**	*Hs.111632*	*LSM3 homolog, U6 small nuclear RNA associated*	*pre-mRNA splicing factor activity*
**LXN**	*Hs.478067*	*latexin*	*enzyme inhibitor activity*
**MTSG1**	*Hs.7946*	*mitochondrial tumor suppressor 1*	*oxidoreductase activity, receptor activity*
**NCOA1**	*Hs.412293*	*nuclear receptor coactivator 1*	*signal transduction*
**NKTR**	*Hs.529509*	*natural killer-tumor recognition sequence*	*protein folding*
**NRF**	*Hs.437084*	*NF-kappaB repressing factor*	*negative regulation of transcription*
**NSE1**	*Hs.260855*	*NSE1*	*unknown function*
**NYREN18**	*Hs.173024*	*NEDD8 ultimate buster-1*	*ubiquitin/proteasome pathway*
**PCTK3**	*Hs.445402*	*PCTAIRE protein kinase 3*	*signal transduction*
**PEX3**	*Hs.7277*	*peroxisomal biogenesis factor 3*	*peroxisome organization and biogenesis*
**POLQ**	*Hs.241517*	*polymerase (DNA directed), theta*	*DNA repair*
**RBM12**	*Hs.246413*	*copine I*	*lipid metabolic process*
**RGS2**	*Hs.78944*	*regulator of G-protein signalling 2*	*cell cycle regulation*
**SF1**	*Hs.502829*	*splicing factor 1*	*regulation of transcription, DNA-dependent*
**SFRS3**	*Hs.405144*	*splicing factor, arginine/serine-rich 3*	*nuclear mRNA splicing, via spliceosome*
**STHM**	*Hs.207459*	*ST6*	*protein amino acid glycosylation*
**SYNJ2**	*Hs.434494*	*synaptojanin 2*	*phosphoinositide 5-phosphatase activity*
**TFPI2**	*Hs.438231*	*tissue factor pathway inhibitor 2*	*serine-type endopeptidase inhibitor activity*
**TFRC**	*Hs.529618*	*transferrin receptor (p90, CD71)*	*endocytosis*
**TP53INP1**	*Hs.492261*	*tumor protein p53 inducible nuclear protein 1*	*apoptosis*
**Unknown**	---------	---------	---------
**ZNF189**	*Hs.50123*	*zinc finger protein 189*	*regulation of transcription, DNA-dependent*

*Genes are listed in alphabetical order. The cutoff p-value was set as $<10^{-9}$.
