# Supplementary figures and images for: Characterization of global transcription profile of normal and HPV-immortalized keratinocytes and their response to TNF treatment
Source: BMC Med Genomics. 2008 Jun 27;1:29. doi: 10.1186/1755-8794-1-29 (PMC2459201; doi:10.1186/1755-8794-1-29)

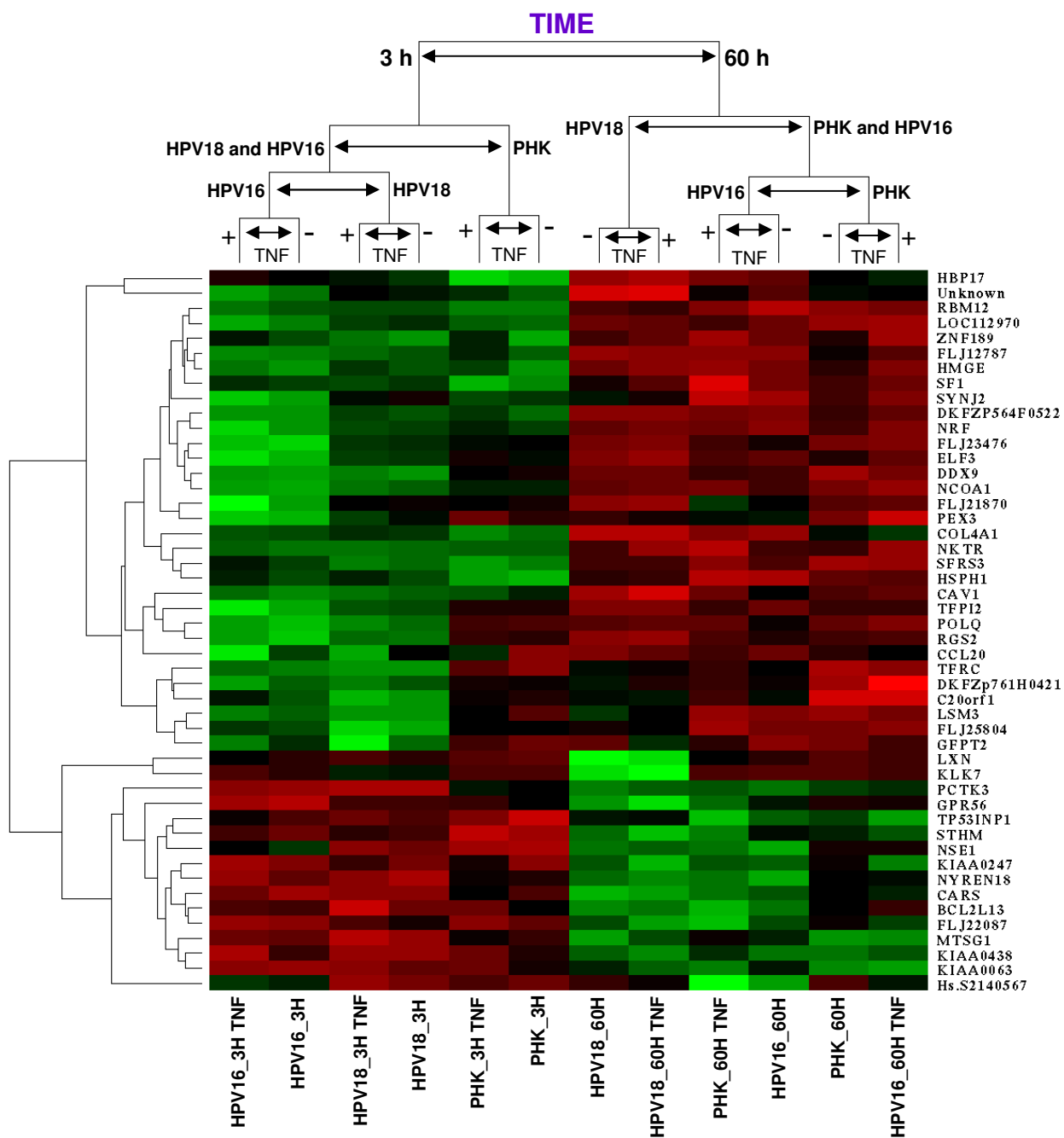

Supplement: Additional file 2 — Hierarchical grouping based on differentially expressed genes as a function of time. These genes where identified by the ANOVA method and the samples where grouped considering the correlation distance and complete linkage. After sample grouping the genes were hierarchically grouped by their correlation distances. High gene expression is shown in red, low gene expression is shown in green and black indicates non-differential gene expression, p values <10-9. Samples: Primary human keratinocytes: controls and treated for 3 or 60 hours with TNF, respectively (PHK_3H, PHK_60H, PHK_3H.TNF, PHK_60H.TNF); HPV16-immortalized keratinocytes: controls and treated for 3 or 60 hours with TNF, respectively (HPV16_3H, HPV16_60H, HPV16_3H.TNF, HPV16_60H.TNF); HPV18-immortalized keratinocytes: controls and treated for 3 or 60 hours with TNF, respectively (HPV18_3H, HPV18_60H, HPV18_3H.TNF, HPV18_60H.TNF). [file 1755-8794-1-29-S2.pdf]

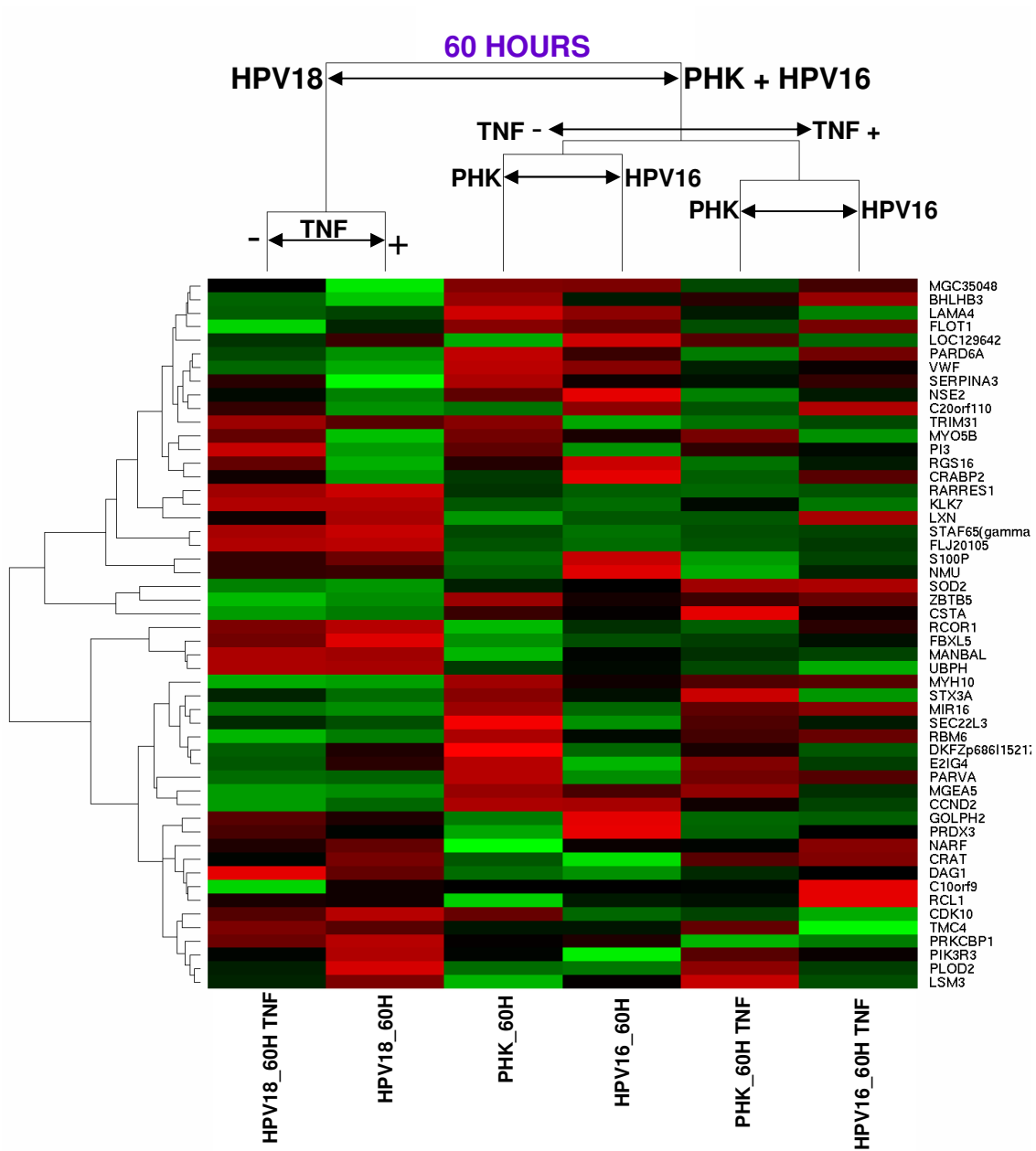

Supplement: Additional file 4 — Supervised hierarchical grouping based on the 30 genes that best differentiate normal and HPV 16-immortalized keratinocytes from the HPV 18-immortalized ones after treatment with TNF for 60 hours. High gene expression is shown in red, low gene expression is shown in red and black indicates non-differential gene expression. PHK_3H (primary human keratinocytes – control/3 hours), PHK_3H TNF (primary human keratinocytes – TNF/3 hours), HPV16_3H (HPV16-immortalized keratinocytes – control/3 hours), HPV16_3H TNF (HPV16-immortalized keratinocytes – TNF/3 hours), HPV18_3H (HPV18-immortalized keratinocytes – control/3 hours), HPV18_3H TNF (HPV18-immortalized keratinocytes – TNF/3 hours). [file 1755-8794-1-29-S4.pdf]

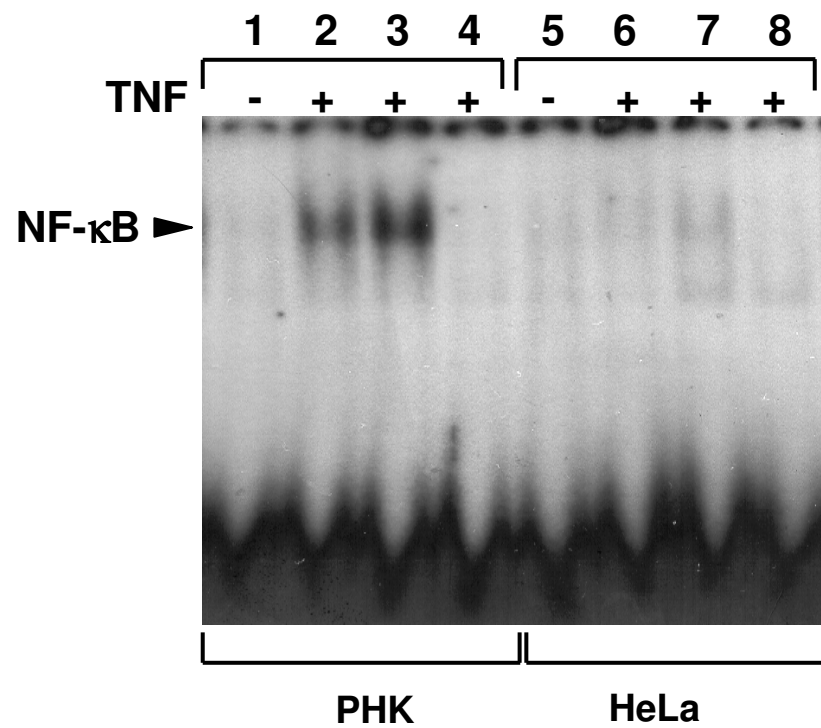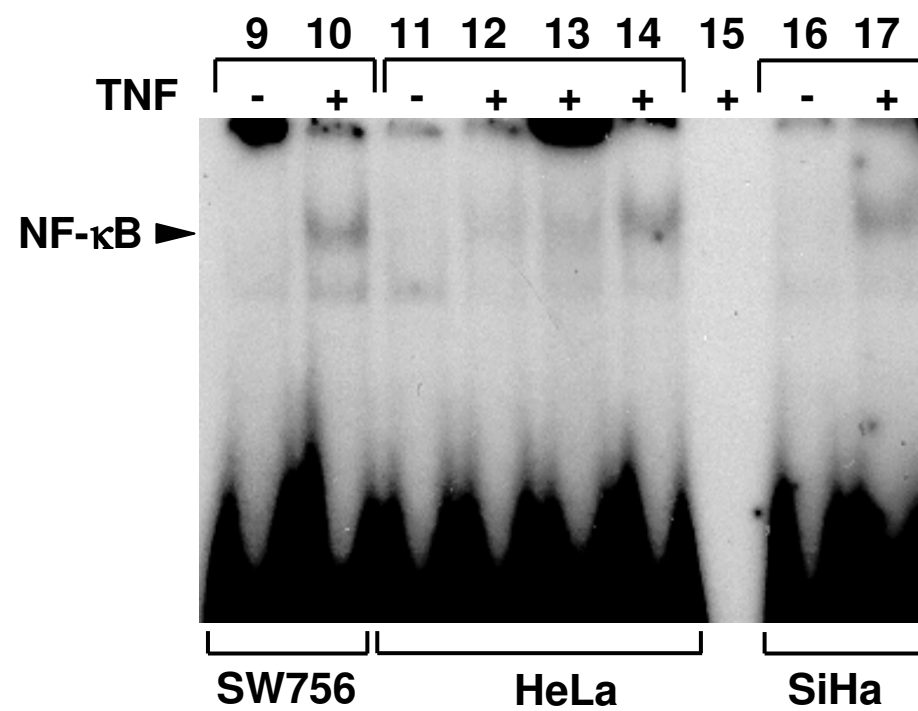

Supplement: Additional file 6 — Analysis of TNF-induced NF-κB activation in normal and HPV16 or 18-transformed keratinocytes. Nuclear protein extracts were obtained from sub confluent cultures of normal keratinocytes (PHK), HPV16-positive (SiHa) and HPV18-positive (SW756 and HeLa) cervical cancer-derived cell lines treated with 2 nM of TNF for 1 h. For each EMSA reaction, 5 mg of nuclear protein were incubated with 50 fmol of [γ-32P]ATP-labeled double-stranded oligonucleotide and a 50X excess of consensus unlabeled oligonucleotide (lanes 4, 8 and 13). Specificity of binding was further demonstrated by incubation of nuclear extracts with the described amount of labeled consensus oligonucleotide and a 50X excess of a labeled oligonucleotide carrying a single-base mutation at the NF-κB binding site (lanes 3, 7 and 14) and incubation of nuclear extract in the absence of any labeled probe (lane 15). NF-κB-DNA binding reactions were carried out as described under "Material and Methods". HeLa nuclear extracts obtained from two different experiments were included as controls of NF-κB activation levels in HPV-positive cell lines. DNA binding complexes are indicated. [file 1755-8794-1-29-S6.pdf]
