# Supplementary material for: Characterization of global transcription profile of normal and HPV-immortalized keratinocytes and their response to TNF treatment
Source: BMC Med Genomics. 2008 Jun 27;1:29. doi: 10.1186/1755-8794-1-29 (PMC2459201; doi:10.1186/1755-8794-1-29)
Supplement: Additional file 3 — Table of differentially expressed genes obtained by pair-wise comparison of all the samples and their respective expression ratio ordered by p-value. PHK_3H (primary human keratinocytes – control/3 hours), PHK_60H (primary human keratinocytes – control/60 hours), HPV16_3H (HPV16-immortalized keratinocytes – control/3 hours), HPV16_60H (HPV16-immortalized keratinocytes – control/60 hours), HPV18_3H (HPV18-immortalized keratinocytes – control/3 hours), HPV18_60H (HPV18-immortalized keratinocytes – control/60 hours), PHK_TNF3H (primary human keratinocytes – TNF/3 hours), PHK_TNF60H (primary human keratinocytes – TNF/60 hours), HPV16_TNF3H (HPV16-immortalized keratinocytes – TNF/3 hours), HPV16_TNF60H (HPV16-immortalized keratinocytes – TNF/60 hours), HPV18_TNF3H (HPV18-immortalized keratinocytes – TNF/3 hours), HPV18_TNF60H (HPV18-immortalized keratinocytes – TNF/60 hours). [file 1755-8794-1-29-S3.pdf]

1ª) PHK_3H / HPV16_3H

p value <0,0001

gene	expression ratio (log_2)	gene name
FLJ11301	-1,78	hypothetical protein FLJ11301
SF3B4	-1,20	splicing factor 3b, subunit 4
B3GNT5	1,74	UDP-GlcNAc:betaGal beta-1,3-N-acetylglucosaminyltransferase 5
KCNK6	3,79	potassium channel, subfamily K, member 6
NCK2	0,45	NCK adaptor protein 2
PGM1	1,81	phosphoglucomutase 1
GLDC	-2,37	glycine dehydrogenase (decarboxylating; glycine decarboxylase, glycine cleavage system protein P)
POLS	1,20	polymerase (DNA directed) sigma
LOC56851	1,90	chromosome 15 open reading frame 24
SLC39A6	1,02	solute carrier family 39 (zinc transporter), member 6
PRSS11	4,27	protease, serine, 11 (IGF binding)
ENC1	1,41	ectodermal-neural cortex (with BTB-like domain)
RER1	1,69	RER1 retention in endoplasmic reticulum 1 homolog
MGAM	-2,09	maltase-glucoamylase (alpha-glucosidase)
ITPKC	-6,60	inositol 1,4,5-trisphosphate 3-kinase C
XTP3TPB	-1,19	chromosome 2 open reading frame 30
RPL15	2,53	ribosomal protein L15
ANKRD11	3,20	ankyrin repeat domain 11
CD44	1,28	CD44 antigen (homing function and Indian blood group system)
SAT2	1,98	solute carrier family 38, member 2
CKLFSF4	-6,59	chemokine-like factor super family 4
SRP72	2,26	signal recognition particle 72kDa
EIF4EBP2	-3,79	eukaryotic translation initiation factor 4E binding protein 2
ZNF262	-0,62	zinc finger protein 262
ZNF266	0,93	zinc finger protein 266
EXTL2	-5,38	exostoses (multiple)-like 2

2ª) PHK_3 H / HPV18_3H

p value <0,0001

gene	expression ratio (log2)	gene name
MAPRE2	-6,89	microtubule-associated protein, RP/EB family, member 2
BCL3	-3,33	B-cell CLL/lymphoma 3
DDX52	-1,90	DEAD (Asp-Glu-Ala-Asp) box polypeptide 52
LCN7	-5,84	lipocalin 7

CNNM3	-6,69	cyclin M3
ITPKC	-6,16	inositol 1,4,5-trisphosphate 3-kinase C
UBE2I	-1,85	ubiquitin-conjugating enzyme E2I (UBC9 homolog, yeast)
KCTD15	-6,30	potassium channel tetramerisation domain containing 15
GIT1	-6,38	G protein-coupled receptor kinase interactor 1
CKLFSF4	-6,26	chemokine-like factor super family 4
UBP1	-1,90	upstream binding protein 1 (LBP-1a)
FLJ10700	-4,33	hypothetical protein FLJ10700
IL6ST	-3,15	interleukin 6 signal transducer (gp130, oncostatin M receptor)
SLPI	-2,80	secretory leukocyte protease inhibitor (antileukoproteinase)
ARF4L	-1,61	ADP-ribosylation factor 4-like
SPAG9	-3,85	sperm associated antigen 9
SURF5	-0,95	surfeit 5
MIR16	0,67	membrane interacting protein of RGS16

3ª) HPV16_3H / HPV18_3H

p value <0,0001

gene	expression ratio (log2)	gene name
C6orf48	-1,47	chromosome 6 open reading frame 48
STAF65(gamma)	-6,16	SPTF-associated factor 65 gamma
TOM1L2	1,03	target of myb1-like 2 (chicken)
SPG20	-1,23	spastic paraplegia 20, spartin (Troyer syndrome)

4ª) PHK_60H / HPV16_60H

p value<0,0001

gene	expression ratio (log2)	gene name
FLJ21511	2,85	hypothetical protein FLJ21511
ROBO1	2,48	roundabout, axon guidance receptor, homolog 1 (Drosophila)
SUPT5H	-1,06	suppressor of Ty 5 homolog (S. cerevisiae)
LACTB2	-1,69	lactamase, beta 2
OAS1	-2,19	2',5'-oligoadenylate synthetase 1, 40/46kDa
TFRC	-1,41	transferrin receptor (p90, CD71)
COX7B	-1,41	cytochrome c oxidase subunit VIIb
C1orf29	-2,15	interferon-induced protein 44
ODC1	2,16	ornithine decarboxylase 1
PPP3CA	1,54	protein phosphatase 3 (formerly 2B), catalytic subunit, alpha isoform (calcineurin A alpha)
PDHB	0,63	pyruvate dehydrogenase (lipoamide) beta
MEST	-1,55	mesoderm specific transcript homolog (mouse)
NT5E	1,77	5'-nucleotidase, ecto (CD73)

TSPYL1	-1,69	TSPY-like 1

5ª) PHK_60H / HPV18_60H

p value <0,0001

gene	expression ratio (log2)	gene name
SCD	-2,09	stearoyl-CoA desaturase (delta-9-desaturase)
CREG	-2,33	cellular repressor of E1A-stimulated genes 1
FAP	-2,45	fibroblast activation protein, alpha
CKLFSF6	2,22	chemokine-like factor super family 6
RUNDC1	-1,18	RUN domain containing 1
DHRS3	-4,22	dehydrogenase/reductase (SDR family) member 3
ODC1	2,13	ornithine decarboxylase 1
LXN	-4,26	latexin
LOC56851	-1,63	chromosome 15 open reading frame 24
MAL2	-1,86	mal, T-cell differentiation protein 2
Ufc1	-1,44	Ufm1-conjugating enzyme 1
CLCA4	2,37	chloride channel, calcium activated, family member 4
LOC129642	-2,15	O-acyltransferase (membrane bound) domain containing 2
ATF7	-3,37	activating transcription factor 7
FLJ34633	-0,65	hypothetical protein FLJ34633
XTP7	-1,38	protein 7 transactivated by hepatitis B virus X antigen (HBxAg)
SLC39A1	-1,99	solute carrier family 39 (zinc transporter), member 1
FLJ21511	1,95	hypothetical protein FLJ21511
ATP6AP1	3,68	ATPase, H+ transporting, lysosomal accessory protein 1
PMP22	-2,71	peripheral myelin protein 22
IL4R	-2,57	interleukin 4 receptor
TSSC1	-1,57	tumor suppressing subtransferable candidate 1
CLIC4	1,47	chloride intracellular channel 4
NDFIP1	1,36	Nedd4 family interacting protein 1
TSPYL1	-2,01	TSPY-like 1
STAF65(gamma)	-6,17	SPTF-associated factor 65 gamma
CUL4A	2,34	cullin 4A
TFRC	-1,53	transferrin receptor (p90, CD71)
ZNF335	-1,74	zinc finger protein 335
CDH18	2,02	cadherin 18, type 2
KIAA1529	-2,23	KIAA1529
SSR4	-2,91	signal seq. receptor, delta (translocon-associated protein delta)
NOTCH2	1,80	Notch homolog 2 (Drosophila)
CENTD1	1,25	centaurin, delta 1
KLK8	-3,04	kallikrein 8 (neuropsin/ovasin)

RER1	-1,25	RER1 retention in endoplasmic reticulum 1 homolog (S. cerevisiae)
ROBO1	2,01	roundabout, axon guidance receptor, homolog 1 (Drosophila)
PRKCBP1	-1,02	protein kinase C binding protein 1
CDK10	-1,66	cyclin-dependent kinase (CDC2-like) 10
MTO1	-2,15	mitochondrial translation optimization 1 homolog (S. cerevisiae)
KCNK6	-2,90	potassium channel, subfamily K, member 6
MYH10	1,00	myosin, heavy polypeptide 10, non-muscle
SLMAP	2,40	sarcolemma associated protein
KPNA6	-1,76	karyopherin alpha 6 (importin alpha 7)
XPO7	-2,20	exportin 7
SLC30A9	-1,54	solute carrier family 30 (zinc transporter), member 9
C6orf37	-2,29	family with sequence similarity 46, member A
RCL1	-0,93	RNA terminal phosphate cyclase-like 1
KIAA0853	0,83	KIAA0853
SLC37A3	-0,68	solute carrier family 37 (glycerol-3-phosphate transporter), member 3
PHLDA3	-1,90	pleckstrin homology-like domain, family A, member 3
P4HB	-4,79	procollagen-proline, 2-oxoglutarate 4-dioxygenase (proline 4-hydroxylase), beta polypeptide (protein disulfide isomerase; thyroid hormone binding protein p55)
MAT2B	2,45	methionine adenosyltransferase II, beta
ABCE1	1,85	ATP-binding cassette, sub-family E (OABP), member 1
HNRPH3	1,53	heterogeneous nuclear ribonucleoprotein H3 (2H9)
COX7B	-1,15	cytochrome c oxidase subunit VIIb
BENE	-2,08	BENE protein
HERC3	0,79	hect domain and RLD 3
SUPT4H1	-1,74	suppressor of Ty 4 homolog 1 (S. cerevisiae)
CASK	-1,38	calcium/calmodulin-dependent serine protein kinase (MAGUK family)

6ª) HPV16_60H / HPV18_60H **p value<0,0001**

gene	expression ratio (log2)	gene name
LXN	-3,82	latexin
C1orf29	2,97	interferon-induced protein 44
STAF65(gamma)	-6,30	SPTF-associated factor 65 gamma
PDHB	-0,72	pyruvate dehydrogenase (lipoamide) beta
LOC129642	-1,27	O-acyltransferase (membrane bound) domain containing
PRKCBP1	-0,86	protein kinase C binding protein 1
YME1L1	-1,66	YME1-like 1 (S. cerevisiae)
XTP3TPB	0,51	chromosome 2 open reading frame 30

7ª) PHK_3H / PHK_60H

p value <0,0001

gene	expression ratio (log2)	gene name
CKLFSF6	-2,27	chemokine-like factor super family 6
PPARA	-3,09	peroxisome proliferative activated receptor, alpha
CKLFSF4	-7,19	chemokine-like factor super family 4
EIF4EBP2	-4,08	eukaryotic translation initiation factor 4E binding protein 2
SCD	1,43	stearoyl-CoA desaturase (delta-9-desaturase)
MTA2	-1,92	metastasis associated 1 family, member 2
RUNDC1	1,24	RUN domain containing 1
KCTD15	-7,23	potassium channel tetramerisation domain containing 15
ROBO1	-2,32	roundabout, axon guidance receptor, homolog 1 (Drosophila)
HMGCR	1,75	3-hydroxy-3-methylglutaryl-Coenzyme A reductase
DFFA	-0,73	DNA fragmentation factor, 45kDa, alpha polypeptide

8ª) HPV16_3H / HPV16_60H

p value <0,0001

gene	expression ratio (log2)	gene name
KIAA0247	-1,42	KIAA0247
NYREN18	-1,78	NEDD8 ultimate buster-1
CD44	0,94	CD44 antigen (homing function and Indian blood group system)
FLJ10761	-1,26	ethanolamine kinase 2
MGC32124	-4,56	hypothetical protein MGC32124
B2M	-2,08	beta-2-microglobulin
TSPYL1	-1,57	TSPY-like 1
PRO1073	-1,37	PRO1073 protein
OCIA	-1,40	OCIA domain containing 1
SURF5	0,91	surfeit 5
BCL2L1	0,93	BCL2-like 1
C14orf130	-1,95	chromosome 14 open reading frame 130
IFI16	-2,00	interferon, gamma-inducible protein 16

9ª) HPV18_3 H/ HPV18_60H

p value <0,0001

gene	expression ratio (log2)	gene name
C6orf48	-1,31	chromosome 6 open reading frame 48
CDK10	-2,09	cyclin-dependent kinase (CDC2-like) 10
LXN	-3,87	latexin
CTSC	2,29	cathepsin C
THBS1	3,21	thrombospondin 1
ABCE1	1,97	ATP-binding cassette, sub-family E (OABP), member 1
CREG	-1,73	Cellular Repressor of E1A-stimulated Genes

HBP17	2,10	fibroblast growth factor binding protein 1
MTO1	-1,48	mitochondrial translation optimization 1 homolog (S. cerevisiae)
UBN1	-1,06	ubinuclein 1
COL4A1	1,68	collagen, type IV, alpha 1
DHRS3	-2,70	dehydrogenase/reductase (SDR family) member 3
Ufc1	-0,93	Ufm1-conjugating enzyme 1
C14orf114	-0,99	chromosome 14 open reading frame 114
C20orf6	1,35	chromosome 20 open reading frame

10ª) PHK_3H / PHK_TNF3H — p value <0,0001

gene	expression ratio (log2)	gene name
CKLFSF4	-6,16	chemokine-like factor super family 4
MAPRE2	-6,88	microtubule-associated protein, RP/EB family, member 2
EIF4EBP2	-3,16	eukaryotic translation initiation factor 4E binding protein 2
ITGA6	-1,57	integrin, alpha 6
PPARA	-2,80	peroxisome proliferative activated receptor, alpha
BCL3	-3,40	B-cell CLL/lymphoma 3
DSC3	-1,78	desmocollin 3
ANKRD11	2,45	ankyrin repeat domain 11
FLJ10700	-4,31	hypothetical protein FLJ10700
NUP205	-1,43	nucleoporin 205kDa
KCTD15	-6,25	potassium channel tetramerisation domain containing 15
CNNM3	-6,68	cyclin M3
C14orf120	-3,65	chromosome 14 open reading frame 120
ITPKC	-6,09	inositol 1,4,5-trisphosphate 3-kinase C
GIT1	-6,29	G protein-coupled receptor kinase interactor 1
LCN7	-5,82	lipocalin 7
SPAG9	-3,81	sperm associated antigen 9
NFKBIA	-2,07	nuclear factor of kappa light polypeptide gene enhancer in B-cells inhibitor, alpha
IL6ST	-3,09	interleukin 6 signal transducer (gp130, oncostatin M receptor)
KLK2	-3,84	kallikrein 2, prostatic
VLDLR	-5,62	very low density lipoprotein receptor

11ª) PHK_60H / PHK_TNF60H — p valor <0,0001

gene	expression ratio (log2)	gene name
MGC33488	0,68	THAP domain containing, apoptosis associated protein 3
MAL2	1,71	mal, T-cell differentiation protein 2
SOD2	-2,42	superoxide dismutase 2, mitochondrial
NFKBIA	-2,30	nuclear factor of kappa light polypeptide gene enhancer in B-cells inhibitor, alpha

12ª) PHK_TNF3H / PHK_TNF60H — p value <0,0001

gene	expression ratio (log_2)	gene name
FLJ21511	-1,83	hypothetical protein FLJ21511
SFRS3	1,59	splicing factor, arginine/serine-rich 3
PP784	-1,29	PP784 protein
RUNDC1	1,87	RUN domain containing 1
PRKDC	0,89	protein kinase, DNA-activated, catalytic polypeptide
ARHGEF10	0,94	Rho guanine nucleotide exchange factor (GEF) 10
HERC3	-0,57	hect domain and RLD 3
SLC30A9	1,10	solute carrier family 30 (zinc transporter), member 9
HBP17	1,26	fibroblast growth factor binding protein 1
FLJ11106	2,24	thioesterase domain containing 1
STK39	-0,96	serine threonine kinase 39 (STE20/SPS1 homolog, yeast)
CA14	1,39	carbonic anhydrase XIV
PPM1B	0,83	protein phosphatase 1B (formerly 2C), magnesium-dependent, beta isoform
TLP19	1,12	endoplasmic reticulum thioredoxin superfamily member, 18 kDa

13ª) HPV16_3H / HPV16_TNF3H — p value <0,0001

gene	expression ratio (log2)	gene name
CCL20	-1,93	chemokine (C-C motif) ligand 20

14ª) HPV16_60H / HPV16_TNF60 — p value <0,0001

gene	expression ratio (log2)	gene name
SOD2	-2,029659266	superoxide dismutase 2, mitochondrial

15ª) HPV16_TNF3H / HPV16_TNF60H — p value <0,0001

gene	expression ratio (log2)	gene name
CCL20	3,27	chemokine (C-C motif) ligand 20
PRKAB1	-1,62	protein kinase, AMP-activated, beta 1 non-catalytic subunit
FLJ20516	1,82	timeless-interacting protein
OAT	2,75	ornithine aminotransferase (gyrate atrophy)
FLJ13213	1,38	hypothetical protein

		FLJ13213
NKTR	1,96	natural killer-tumor recognition sequence
SIMP	-0,80	source of immunodominant MHC-associated peptides
DKFZP564F0522	1,45	DKFZP564F0522 protein
PMSCL1	0,98	exosome component 9
CETN3	1,42	centrin, EF-hand protein, 3 (CDC31 homolog, yeast)
KPNB3	1,05	RAN binding protein 5

16ª) HPV18_3H / HPV18_TNF3H **p value <0,01**

gene	expression ratio (log2)	gene name
CCL20	-1,12	chemokine (C-C motif) ligand 20
PCBP1	0,17	poly(rC) binding protein 1

17ª) HPV18_60H / HPV18_TNF60H **p value <0,0001**

gene	expression ratio (log2)	gene name
LAMA3	-1,42	laminin, alpha 3
ALDH1A3	-1,75	aldehyde dehydrogenase 1 family, member A3

18ª) HPV18_TNF3H / HPV18_TNF60H **p value <0,0001**

gene	expression ratio (log2)	gene name
CCL20	2,13	chemokine (C-C motif) ligand 20
SCA2	-0,61	ataxin 2-binding protein 1
HYPK	1,19	Huntingtin interacting protein K
LXN	-3,21	latexin
NSE1	-1,29	NSE1
ATP6AP1	1,87	ATPase, H+ transporting, lysosomal accessory protein 1
CREG	-1,64	cellular repressor of E1A-stimulated genes 1
C20orf6	1,54	chromosome 20 open reading frame 6
PCTK3	-3,19	PCTAIRE protein kinase 3
HBP17	2,25	fibroblast growth factor binding protein 1
CARS	-1,09	cysteinyl-tRNA synthetase
UBN1	-1,20	ubinuclein 1
GPR56	-2,30	G protein-coupled receptor 56
DHX9	1,04	DEAH (Asp-Glu-Ala-His) box polypeptide 9
COL4A1	1,38	collagen, type IV, alpha 1
SIAT7B	-1,82	sialyltransferase 7 ((alpha-N-acetylneuraminyl-2,3-beta-galactosyl-1,3)-N-acetyl galactosaminide alpha-2,6-sialyltransferase) B
LBR	1,07	lamin B receptor

ZNF189	1,17	NO QUERY
UBE2L6	-1,26	ubiquitin-conjugating enzyme E2L 6
MGEA5	1,26	meningioma expressed antigen 5 (hyaluronidase)

19ª) PHK_TNF3H / HPV16_TNF3H **p value <0,0001**

gene	expression ratio (log2)	gene name
KIAA0310	1,18	KIAA0310
TGFB1I4	2,37	transforming growth factor beta 1 induced transcript 4
ARHGEF10	2,71	Rho guanine nucleotide exchange factor (GEF) 10
HBP17	2,28	fibroblast growth factor binding protein 1
TPBG	2,42	trophoblast glycoprotein
HMGCR	1,90	3-hydroxy-3-methylglutaryl-Coenzyme A reductase
PRSS11	4,71	protease, serine, 11 (IGF binding)
FLJ11106	2,55	thioesterase domain containing 1
PPM1B	1,61	protein phosphatase 1B (formerly 2C), magnesium-dependent, beta isoform
FLJ11588	2,84	hypothetical protein FLJ11588
EIF3S1	1,73	eukaryotic translation initiation factor 3, subunit 1 alpha, 35kDa
LOC133619	1,92	hypothetical protein MGC12103
ITGA6	3,31	integrin, alpha 6
PLAU	2,49	plasminogen activator, urokinase
RER1	1,62	RER1 retention in endoplasmic reticulum 1 homolog (S. cerevisiae)
SIMP	1,68	source of immunodominant MHC-associated peptides
SF3B4	-1,83	splicing factor 3b, subunit 4, 49kDa
RNF130	1,88	ring finger protein 130
TMC4	1,99	transmembrane channel-like 4
FKBP5	2,06	FK506 binding protein 5
TMEM2	-0,64	transmembrane protein 2
PRO2730	1,54	hypothetical protein PRO2730
LOC56851	2,14	chromosome 15 open reading frame 24
NICE-3	3,94	chromosome 1 open reading frame 43
PEX3	-1,31	peroxisomal biogenesis factor 3
CTCF	2,41	CCCTC-binding factor (zinc finger protein)
PRKAB1	1,36	protein kinase, AMP-activated, beta 1 non-catalytic subunit
SAT2	2,28	spermidine/spermine N1-acetyltransferase 2
FDFT1	4,30	farnesyl-diphosphate farnesyltransferase 1
PRO1073	1,15	PRO1073 protein
ABR	-0,62	active BCR-related gene
E2IG5	-1,35	growth and transformation-dependent protein
GC20	1,64	translation factor sui1 homolog
ENC1	1,15	ectodermal-neural cortex (with BTB-like domain)

ENAH	-2,69	enabled homolog (Drosophila)
FLJ21511	1,35	hypothetical protein FLJ21511
LAMA3	2,87	laminin, alpha 3
SUPV3L1	1,61	suppressor of var1, 3-like 1 (S. cerevisiae)
PARG	-2,13	poly (ADP-ribose) glycohydrolase
LIMK2	1,36	LIM domain kinase 2
DJ167A19.1	1,74	hypothetical protein DJ167A19.1
CCL20	-1,86	chemokine (C-C motif) ligand 20
OSR1	4,38	odd-skipped related 1 (Drosophila)
ZFP90	1,60	zinc finger protein 90 homolog (mouse)
ACN9	-1,22	ACN9 homolog (S. cerevisiae)
OAT	-2,77	ornithine aminotransferase (gyrate atrophy)
PGM1	1,64	phosphoglucomutase 1
H41	2,24	hypothetical protein H41
MAP4	1,45	microtubule-associated protein 4
POGZ	-1,68	pogo transposable element with ZNF domain
VAMP3	1,25	vesicle-associated membrane protein 3 (cellubrevin)
PPP4R1	2,48	protein phosphatase 4, regulatory subunit 1
LOC84661	-1,01	dpy-30-like protein
CUL1	1,90	cullin 1
C20orf111	-0,93	chromosome 20 open reading frame 111
ALDH1A3	1,73	aldehyde dehydrogenase 1 family, member A3
DKFZp434D0215	1,08	SH3 domain protein D19
KIAA0494	-0,68	KIAA0494 gene product
GSR	-1,06	glutathione reductase
MAL2	-3,13	mal, T-cell differentiation protein 2
CD44	1,14	CD44 antigen (homing function and Indian blood group system)
OAS1	-1,24	2',5'-oligoadenylate synthetase 1, 40/46kDa
LACTB2	-1,86	lactamase, beta 2
LCP1	1,78	lymphocyte cytosolic protein 1 (L-plastin)
ECT2	-1,66	epithelial cell transforming sequence 2 oncogene
D4S234E	1,18	DNA segment on chromosome 4 (unique) 234 expressed sequence
PP784	0,86	PP784 protein
CDC42BPA	-0,92	CDC42 binding protein kinase alpha (DMPK-like)
FLJ20073	-0,99	sterile alpha motif domain containing 9
RBM3	-1,50	RNA binding motif (RNP1, RRM) protein 3
PPIB	3,62	peptidylprolyl isomerase B (cyclophilin B)
LITAF	2,10	lipopolysaccharide-induced TNF factor
ARF4	1,39	ADP-ribosylation factor 1
NCOA1	-0,80	nuclear receptor coactivator 1
FEM1C	0,99	fem-1 homolog c (C.elegans)
MGC45714	-1,05	chromosome 17 open reading frame 32
ATF7	4,03	activating transcription factor 7
GPS1	-0,54	G protein pathway suppressor 1

DKFZP586N0721	2,26	SMAD, mothers against DPP homolog 3 (Drosophila
KIAA1671	-0,67	KIAA1671 protein
YWHAH	-0,81	tyrosine 3-monooxygenase/tryptophan 5-monooxygenase activation protein, eta polypeptide
HNRPA2B1	-1,18	heterogeneous nuclear ribonucleoprotein A2/B1
MYH10	-0,72	myosin, heavy polypeptide 10, non-muscle
ARID1B	2,70	AT rich interactive domain 1B (SWI1-like)
FLJ13089	-1,07	hypothetical protein FLJ13089
MRPS15	3,16	mitochondrial ribosomal protein S15

20ª) PHK_TNF3 H / HPV18_TNF3H **p value <0,0001**

gene	expression ratio (log2)	gene name
PRSS11	4,75	protease, serine, 11 (IGF binding)
MAL2	-3,76	mal, T-cell differentiation protein 2
SF3B4	-2,04	splicing factor 3b, subunit 4, 49kDa
HBP17	1,65	fibroblast growth factor binding protein 1
NT5E	2,06	5'-nucleotidase, ecto (CD73)
INPP4B	-1,14	inositol polyphosphate-4-phosphatase, type II, 105kDa
HEXB	-0,62	hexosaminidase B (beta polypeptide)
SLC41A3	0,68	solute carrier family 41, member 3
YME1L1	-1,39	YME1-like 1 (S. cerevisiae)
ODC1	1,39	ornithine decarboxylase 1
STAF65(gamma)	-4,79	SPTF-associated factor 65 gamma
ARNT2	1,09	aryl-hydrocarbon receptor nuclear translocator 2
MGC45400	0,78	transcription elongation factor A (SII)-like 8

21ª) HPV16_TNF3 H / HPV18_TNF3H **p value <0,0001**

gene	expression ratio (log2)	gene name
OAS1	1,72	2',5'-oligoadenylate synthetase 1, 40/46kDa
STAF65(gamma)	-5,54	SPTF-associated factor 65 gamma
LOC84661	0,92	dpy-30-like protein
C6orf48	-1,39	chromosome 6 open reading frame 48

22ª) PHK_TNF60H / HPV16_TNF60H **p value <0,0001**

gene	expression ratio (log2)	gene name
MAL2	-2,24	mal, T-cell differentiation protein 2
TCERG1	1,29	transcription elongation regulator 1

ESDN	3,30	discoidin, CUB and LCCL domain containing 2
MGC33488	-1,63	THAP domain containing, apoptosis associated protein 3
DOCK9	2,28	dedicator of cytokinesis 9
CITED1	-1,21	Cbp/p300-interacting transactivator with Glu/Asp-rich carboxy-terminal domain1
SLMAP	2,54	sarcolemma associated protein
TACR2	-0,53	tachykinin receptor 2
PRSS11	3,38	protease, serine, 11 (IGF binding)
NOTCH2	1,50	Notch homolog 2 (Drosophila)
FNBP3	-0,54	formin binding protein 3
HBP17	2,12	fibroblast growth factor binding protein 1
FKBP5	1,67	FK506 binding protein 5
TPRT	1,56	trans-prenyltransferase
PPGB	-1,26	protective protein for beta-galactosidase (galactosialidosis)

23ª) PHK_TNF60H / HPV18_TNF60H **p value <0,0001**

gene	expression ratio (log2)	gene name
MYO5B	-2,54	myosin VB
MAL2	-4,50	mal, T-cell differentiation protein 2
GPR56	-2,13	G protein-coupled receptor 56
LXN	-3,12	latexin
MGC45400	0,85	transcription elongation factor A (SII)-like 8
MRPS35	-1,93	mitochondrial ribosomal protein S35
FLJ21511	2,31	hypothetical protein FLJ21511
SF3B4	-2,12	splicing factor 3b, subunit 4, 49kDa
RUNDC1	-1,98	RUN domain containing 1
MEST	-1,06	mesoderm specific transcript homolog (mouse)
PRSS11	4,89	protease, serine, 11 (IGF binding)
CSTA	3,83	cystatin A (stefin A)
NMES1	-3,79	normal mucosa of esophagus specific 1
GCAT	-1,73	glycine C-acetyltransferase (2-amino-3-ketobutyrate coenzyme A ligase)
RAB31	2,68	RAB31, member RAS oncogene family
SLC30A9	-1,91	solute carrier family 30 (zinc transporter), member 9
DHRS3	-4,01	dehydrogenase/reductase (SDR family) member 3
FKBP5	2,08	dehydrogenase/reductase (SDR family) member 3
PMP22	-2,05	peripheral myelin protein 22
ESDN	2,62	discoidin, CUB and LCCL domain containing 2
DJ167A19.1	-1,60	hypothetical protein DJ167A19.1
SNAI2	2,82	snail homolog 2 (Drosophila)
RER1	-0,93	RER1 retention in endoplasmic reticulum 1 homolog (S. cerevisiae)

CLCA4	2,23	chloride channel, calcium activated, family member 4
SEC61A1	-2,20	Sec61 alpha 1 subunit (S. cerevisiae)
CITED1	-1,23	Cbp/p300-interacting transactivator with Glu/Asp-rich carboxy-terminal domain1
KCNK6	-2,71	potassium channel, subfamily K, member 6
ZMPSTE24	-2,07	zinc metallopeptidase (STE24 homolog, yeast
STAF65(gamma)	-6,09	SPTF-associated factor 65 gamma
CKLFSF6	2,05	chemokine-like factor super family 6
TRAM1	-2,24	translocation associated membrane protein 1
RANGAP1	-1,57	RAN binding protein 1
SLMAP	1,61	sarcolemma associated protein
NSE2	-1,61	breast cancer membrane protein 101
C10orf76	-3,46	chromosome 10 open reading frame 76
MGEA5	2,19	meningioma expressed antigen 5 (hyaluronidase)
SOD2	2,09	superoxide dismutase 2, mitochondrial
ADORA2B	-2,32	adenosine A2b receptor
PPIB	-3,00	peptidylprolyl isomerase B (cyclophilin B)
CHAF1A	1,69	chromatin assembly factor 1, subunit A (p150)
SPPL3	-0,85	signal peptide peptidase 3
C6orf37	-2,09	family with sequence similarity 46, member A
NT5E	2,58	5'-nucleotidase, ecto (CD73)
LCN2	-5,11	lipocalin 2 (oncogene 24p3)
MYO10	2,04	myosin X
PARVA	1,41	parvin, alpha
GBF1	-3,12	golgi-specific brefeldin A resistance factor 1
ZF	0,85	HCF-binding transcription factor Zhangfei
UCHL3	-1,37	ubiquitin carboxyl-terminal esterase L3 (ubiquitin thiolesterase)
HBP17	2,63	fibroblast growth factor binding protein 1
JMJD1B	1,12	jumonji domain containing 1B
CSNK1A1	1,89	casein kinase 1, alpha 1
GOLPH2	-1,47	golgi phosphoprotein 2
ENAH	1,66	enabled homolog (Drosophila)
EPS8L1	-2,29	EPS8-like 1
FAP	-2,84	fibroblast activation protein, alpha
NOTCH2	1,52	Notch homolog 2 (Drosophila)
ENTH	-1,84	enthoprotin
PRKDC	-1,53	protein kinase, DNA-activated, catalytic polypeptide
LOC51204	-1,68	clone HQ0477 PRO0477p
C6orf48	-1,61	chromosome 6 open reading frame 48
NICE-3	-1,84	chromosome 1 open reading frame 43
HAX1	-4,57	HS1 binding protein
Ufc1	-1,24	Ufm1-conjugating enzyme 1
CAPG	-3,47	capping protein (actin filament), gelsolin-like
CREG	-2,35	cellular repressor of E1A-stimulated genes 1

PRKCBP1	-1,18	protein kinase C binding protein 1
LACTB2	-1,50	lactamase, beta 2
NYREN18	-0,65	ultimate buster-1
SSR4	-2,53	signal sequence receptor, delta (translocon-associated protein delta)
TES	-2,56	testis derived transcript (3 LIM domains)
B3GNT5	-1,90	UDP-GlcNAc:betaGal beta-1,3-N-acetylglucosaminyltransferase 5
HIRIP5	-1,19	HIRA interacting protein 5
MTO1	-2,10	mitochondrial translation optimization 1 homolog (S. cerevisiae)
ZNF38	0,65	zinc finger protein 38
CDK5RAP2	-1,10	CDK5 regulatory subunit associated protein 2
C1orf25	1,19	chromosome 1 open reading frame 25
SEC22L3	-0,95	SEC22 vesicle trafficking protein-like 3 (S. cerevisiae)
TSSC1	-1,08	tumor suppressing subtransferable candidate 1
ATP6AP1	3,47	ATPase, H+ transporting, lysosomal accessory protein 1
ATF7	-3,10	activating transcription factor 7
SH3KBP1	-1,08	SH3-domain kinase binding protein 1
MAP1B	-0,71	microtubule-associated protein 1B
AASDHPPT	-1,11	aminoadipate-semialdehyde dehydrogenase-phosphopantetheinyl transferase
TH1L	-2,57	TH1-like (Drosophila)
IL4R	-1,65	interleukin 4 receptor
WDR13	-1,29	WD repeat domain 13
MCM2	-1,67	MCM2 minichromosome maintenance deficient 2, mitotin (S. cerevisiae)
TACSTD1	-1,03	tumor-associated calcium signal transducer 1

24ª) HPV16_TNF60H / HPV18_TNF60H **p value <0,0001**

gene	expression ratio (log2)	gene name
MYO5B	-2,88	myosin VB
SOD2	2,50	superoxide dismutase 2, mitochondrial
MAL2	-2,25	mal, T-cell differentiation protein 2
STAF65(gamma)	-6,02	SPTF-associated factor 65 gamma
ERAL1	-0,90	Era G-protein-like 1 (E. coli)
ATP6AP1	1,12	ATPase, H+ transporting, lysosomal accessory protein 1
GOLPH2	-1,46	golgi phosphoprotein 2
SLC30A9	-1,04	solute carrier family 30 (zinc transporter), member 9
RPL15	-2,10	ribosomal protein L15
NMES1	-1,90	normal mucosa of esophagus specific 1
VMP1	1,05	likely ortholog of rat vacuole membrane protein 1
