# Supplementary material for: Characterization of global transcription profile of normal and HPV-immortalized keratinocytes and their response to TNF treatment
Source: BMC Med Genomics. 2008 Jun 27;1:29. doi: 10.1186/1755-8794-1-29 (PMC2459201; doi:10.1186/1755-8794-1-29)
Supplement: Additional file 5 — Table of differentially expressed genes that best distinguish TNF-resistant (HPV18) from TNF-sensitive cells (PHK and HPV16), in normal culture conditions or upon treatment with TNF for 60 hours. [file 1755-8794-1-29-S5.pdf]

GENE	GENE NAME	(PHK and HPV16) *vs* HPV18		(PHK_TNF and HPV16_TNF) *vs* HPV18_TNF	
		FOLD	p VALUE	FOLD	p VALUE
**BHLHB3**	*basic helix-loop-helix domain containing, class B, 3*	----	----	0.44408	1.2e-05
**C10orf9**	*Cyclin Y*	0.605839	0.000247	----	----
**C20orf110**	*tumor protein p53 inducible nuclear protein 2*	----	----	0.374386	1.9e-05
**CCND2**	*cyclin D2*	2.957399	9.6e-05	----	----
**CDK10**	*cyclin-dependent kinase (CDC2-like) 10*	0.285227	0.000125	----	----
**CRABP2**	*cellular retinoic acid binding protein 2*	----	----	0.276522	2.2e-05
**CRAT**	*carnitine acetyltransferase*	0.343795	1e-06	----	----
**CSTA**	*cystatin A (stefin A)*	2.864497	1.6e-05	----	----
**DAG1**	*dystroglycan 1*	0.474152	0.000138	0.329814	3e-06
**DKFZp686I15217**	*DKFZp686I15217*	0.507912	7.4e-05	----	----
**E2IG4**	*likely ortholog of chicken tsukushi*	0.554887	2.5e-05	----	----
**FBXL5**	*F-box and leucine-rich repeat protein 5*	0.291319	0.000167	----	----
**FLJ20105**	*FLJ20105*	0.01189	0	0.010515	0
**FLOT1**	*flotillin 1*	0.384462	7.6e-05	----	----
**GOLPH2**	*golgi phosphoprotein 2*	----	----	0.36294	0
**KLK7**	*kallikrein 7 (chymotryptic, stratum corneum)*	0.059733	0	0.047716	0
**LAMA4**	*laminin, alpha 4*	----	----	0.320101	2e-06
**LOC129642**	*O-acyltransferase (membrane bound)domain containing 2*	0.305784	7e-05	0.362908	8e-06
**LSM3**	*LSM3 homolog, U6 small nuclear RNA associated (S. cerevisiae)*	0.532545	5.9e-05	----	----
**LXN**	*latexin*	0.06076	0	----	----
**MANBAL**	*mannosidase, beta A, lysosomal-like*	----	----	0.370772	6e-06
**MGC35048**	*hypothetical protein MGC35048*	----	----	0.27868	0
**MGEA5**	*meningioma expressed antigen 5 (hyaluronidase)*	3.402304	6e-06	----	----
**MIR16**	*membrane interacting protein of RGS16*	0.662855	7e-05	----	----
**MYH10**	*encoding myosin, heavy polypeptide 10, non-muscle*	1.836818	0.000209	----	----
**MYO5B**	*acetyl-Coenzyme A acyltransferase 2*	----	----	0.152971	0
**NARF**	*nuclear prelamin A recognition factor*	0.256847	7.6e-05	----	----
**NMU**	*neuromedin U*	----	----	0.058214	1e-06
**NSE2**	*breast cancer membrane protein 101*	----	----	0.348974	2e-06
**PARD6A**	*par-6 partitioning defective 6 homolog alpha (C.elegans)*	----	----	0.39875	2.1e-05
**PARVA**	*parvin, alpha*	----	----	2.560157	2.1e-05
**PI3**	*protease inhibitor 3, skin-derived (SKALP)*	----	----	0.096425	0
**PIK3R3**	*phosphoinositide-3-kinase, regulatory subunit, polypeptide 3 (p55, gamma)*	0.586743	9.9e-05	----	----
**PLOD2**	*procollagen-lysine*	0.455836	0.000133	----	----
**PRDX3**	*peroxiredoxin 3*	----	----	0.422824	3.2e-05

**PRKCBP1**	*protein kinase C binding protein 1*	0.520862	0	0.450365	2e-06
**RARRES1**	*retinoic acid receptor responder*	0.029146	0	0.027975	0
**RBM6**	*RNA binding motif protein 6*	0.792829	0.000193	----	----
**RCL1**	*terminal phosphate cyclase-like 1*	0.592469	0.000133	----	----
**RCOR1**	*REST corepressor 1*	0.170143	0.000262	----	----
**RGS16**	*regulator of G-protein signalling 16*	----	----	0.126675	6e-06
**S100P**	*S100 calcium binding protein P*	0.053016	1.5e-05	0.03839	0
**SEC22L3**	*SEC22 vesicle trafficking protein-like 3 (S. cerevisiae)*	----	----	0.486028	1.2e-05
**SERPINA3**	*serine (or cysteine) proteinase inhibitor*	----	----	0.300802	3e-06
**SOD2**	*superoxide dismutase 2*	----	----	4.901559	0
**STAF65 (gamma)**	*SPTF-associated factor 65 gamma*	0.013265	0	0.015004	0
**STX3A**	*olfactory receptor, family 10, subfamily Y*	0.688883	0.00019	----	----
**TMC4**	*transmembrane channel-like 4*	0.492301	5.1e-05	----	----
**TRIM31**	*tripartite motif-containing 31*	----	----	0.178779	0
**UBPH**	*similar to ubiquitin binding protein*	----	----	0.437032	6e-06
**VWF**	*von Willebrand factor*	----	----	0.485927	2.2e-05
**ZBTB5**	*zinc finger and BTB domain containing 5*	----	----	4.239272	2.6e-05

*Genes are listed in alphabetical order. Underlined genes were identified as differentially expressed between TNF sensitive and TNF resistant cells in both culture conditions.
